# Cybersecurity in Radio Frequency Technologies: A Scientometric and Systematic Review with Implications for IoT and Wireless Applications

**DOI:** 10.3390/s26020747

**Published:** 2026-01-22

**Authors:** Patrícia Rodrigues de Araújo, José Antônio Moreira de Rezende, Décio Rennó de Mendonça Faria, Otávio de Souza Martins Gomes

**Affiliations:** 1 Systems Engineering and Information Technology Institute, Federal University of Itajubá (UNIFEI), Itajubá 37500-903, MG, Brazil; jose.rezende@ifmg.edu.br (J.A.M.d.R.); deciorenno@unifei.edu.br (D.R.d.M.F.); otavio.gomes@unifei.edu.br (O.d.S.M.G.); 2 Academic Area of Electrical Engineering, Federal Institute of Minas Gerais (IFMG), Formiga 35577-020, MG, Brazil

**Keywords:** cybersecurity, radio frequency (RF), wireless communication, scientometric analysis, internet of things (IoT)

## Abstract

Cybersecurity in radio frequency (RF) technologies has become a critical concern, driven by the expansion of connected systems in urban and industrial environments. Although research on wireless networks and the Internet of Things (IoT) has advanced, comprehensive studies that provide a global and integrated view of cybersecurity development in this field remain limited. This work presents a scientometric and systematic review of international publications from 2009 to 2025, integrating the PRISMA protocol with semantic screening supported by a Large Language Model to enhance classification accuracy and reproducibility. The analysis identified two interdependent axes: one focusing on signal integrity and authentication in GNSS systems and cellular networks; the other addressing the resilience of IoT networks, both strongly associated with spoofing and jamming, as well as replay, relay, eavesdropping, and man-in-the-middle (MitM) attacks. The results highlight the relevance of RF cybersecurity in securing communication infrastructures and expose gaps in widely adopted technologies such as RFID, NFC, BLE, ZigBee, LoRa, Wi-Fi, and unlicensed ISM bands, as well as in emerging areas like terahertz and 6G. These gaps directly affect the reliability and availability of IoT and wireless communication systems, increasing security risks in large-scale deployments such as smart cities and cyber–physical infrastructures.

## 1. Introduction

The growing adoption of digital systems in critical infrastructures and urban ecosystems has intensified the global dependence on wireless communication systems [1,2,3,4,5]. In this context, cybersecurity in radio frequency (RF) technologies emerges as a strategic and interdisciplinary field, essential for strengthening the reliability of applications in the Internet of Things (IoT), Smart Cities, and Cyber–Physical Systems [6,7,8,9,10]. As connected devices take on vital functions in areas such as mobility, energy, healthcare, and public safety, the vulnerabilities of the electromagnetic spectrum become a critical factor for the technological resilience and digital sovereignty of nations and institutions [11,12,13,14,15]. Recent studies further emphasize that RF-based attacks can generate systemic risks for wireless and cyber–physical systems, particularly in safety-critical and mobility-oriented applications, reinforcing the need for structured frameworks to analyze vulnerabilities and mitigation strategies across communication layers [16,17].

In recent years, systematic reviews and scientometric studies have proven essential for understanding the evolution of emerging scientific fields and identifying relevant research gaps in cybersecurity [18,19,20,21,22]. Studies such as [23], which conducted a systematic review on radio frequency threats in connected medical devices (IoMT), highlight the severity of vulnerabilities in sensitive contexts such as digital health. Complementarily, refs. [24,25,26] conducted broad scientometric investigations on the Internet of Things, demonstrating the central role of RF based technologies in the expansion of the connected device ecosystem and the corresponding increase in attack surfaces.

Despite the advances achieved and the current stage of research, there is still no globally scoped study specifically dedicated to cybersecurity in radio frequency technologies. The literature remains fragmented, distributed across sectoral investigations and approaches limited to specific technologies or protocols [23,24,27], which hinders the formulation of an integrated view of the state of the art and the main trends in the field. Furthermore, there is a noticeable lack of comprehensive quantitative analyses capable of systematizing the evolution of scientific production on the topic, encompassing dimensions such as temporal growth, publication impact, institutional collaborations, and thematic areas.

In light of this scenario, the present article proposes a global scientometric analysis of research on RF technology cybersecurity. The study systematizes and examines publications indexed in international databases with the objective of providing a detailed view of the temporal dynamics, scientific impact, collaboration networks, and research trends that characterize this domain. By identifying patterns of production and co-authorship, as well as areas of specialization and growth, this work contributes to mapping and understanding the scientific evolution of RF cybersecurity, offering valuable insights for advancing new investigations and strengthening digital resilience in connected communication ecosystems.

Although this study provides a global scientometric perspective on cybersecurity in radio frequency technologies, many of the technologies and threats examined, such as spoofing, jamming, replay, relay, and eavesdropping, have direct implications for wireless embedded systems, IoT devices, smart infrastructure, and low-power wireless networks. To address this, this study combines scientometric analysis with the classification of cyberattack types across different RF-based wireless technologies. This integrative approach helps to identify cybersecurity priorities for IoT environments and wireless applications that rely on RF technologies, providing a foundation for future studies aimed at developing more resilient and secure communication architectures.

In this context, the main contribution of this study lies in strengthening existing and emerging cybersecurity frameworks for RF-based systems by providing a structured and evidence-based overview of how vulnerabilities, attack types, and affected technologies are distributed across the literature. By synthesizing fragmented research into a coherent scientometric and systematic perspective, the study helps clarify where current RF security frameworks are well supported by evidence and where significant gaps remain for IoT, wireless communications, and cyber–physical systems.

Unlike prior review studies that primarily address specific protocols, application domains, or individual attack classes, this work adopts an explicitly RF-centric perspective and integrates large-scale scientometric analysis with systematic attack classification across heterogeneous wireless technologies. This combination enables a cross-domain view of RF cybersecurity that goes beyond isolated technological contexts and is not commonly articulated in existing surveys.

Based on these objectives, the following research questions (RQs) were formulated to guide the analysis and systematic mapping of the field:

RQ1: How has the scientific production of cybersecurity in RF technologies evolved during the period from 2009 to 2025?
RQ2: Which countries, institutions, authors, and collaboration networks stand out as the main contributors to the advancement of the field?
RQ3: Which journals and articles exhibit the highest impact and influence in consolidating research on RF technology cybersecurity?
RQ4: What thematic and technological trends are shaping the future of research in RF cybersecurity?
RQ5: Which types of RF-based attacks have received greater attention in the scientific literature?
RQ6: Which RF technologies are most frequently associated with cybersecurity research?

## 2. Materials and Methods

The methodology of this study combines classical and well-established principles of systematic review and scientometric analysis with automated screening techniques based on Large Language Models (LLMs). The process was designed in accordance with the PRISMA 2020 protocol (Preferred Reporting Items for Systematic Reviews and Meta-Analyses) [28], using the Bibliometrix v5.1.1 package [29] for R language v4.4.3 and a Python v3.12.3 script integrated with the OpenAI API with the GPT-4o-mini model for the semantic screening stage assisted by artificial intelligence (AI) [30,31,32,33,34].

The overall methodological process comprised the following main steps:

1. Formulation of the search strategy and construction of the query strings;
2. Data collection from the Scopus and Web of Science databases;
3. Standardization and deduplication using Bibliometrix;
4. Preliminary manual screening;
5. Automated semantic screening via LLM;
6. Human-in-the-loop validation.

**Figure 1:**
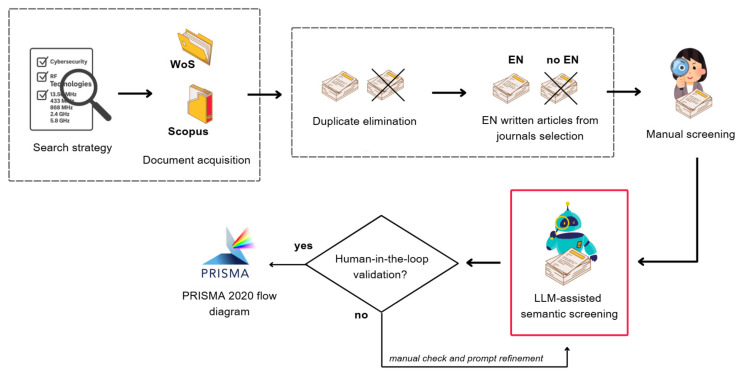
Procedures for article acquisition and screening according to the PRISMA 2020 protocol, including the semantic screening assisted by LLM (highlighted in red).

Figure 1 provides an overview of the methodological flow, highlighting the integration between the traditional review stages and the AI-assisted semantic classification process.

This methodological integration is justified by the efficiency suggested in recent studies on the use of LLMs for automated screening and by the need to handle large volumes of scientific publications [35,36]. Evidence indicates that incorporating LLMs into systematic reviews can reduce analysis time and increase accuracy while maintaining the traceability of results [32,37,38].

The following subsections detail the methodological procedure adopted, covering the stages of search, acquisition, screening, and LLM-assisted validation.

### 2.1. Search Strategy and Article Acquisition

The formulation of the search strategy aimed to identify publications that address the intersection between cybersecurity and radio frequency communication technologies. This stage is methodologically critical, as it defines the scope, representativeness, and reproducibility of the corpus, reinforcing the consistency of the scientometric results and the transparency of the review process [29,39].

The search string was structured into three conceptual blocks:

- Cybersecurity and threat-related terms, encompassing cybersecurity, attack, vulnerability, threat, spoofing, replay, eavesdropping, man-in-the-middle, jamming, among others;
- RF technologies and protocols, including Bluetooth, BLE, ZigBee, Wi-Fi, LoRa, RFID, NFC, NB-IoT, LTE, 5G, GNSS, V2X, DSRC, UWB, and Satellite IoT, among others;
- Frequency bands used as technical descriptors of technologies without explicit citation of a protocol (13.56 MHz, 433 MHz, 868 MHz, 2.4 GHz, 5.8 GHz, 24 GHz, 76 GHz, 81 GHz), among others.

To minimize potential evaluation biases, semantic proximity operators (W/n in Scopus and NEAR/n in Web of Science) were applied to restrict the inclusion of generic frequency unit mentions only when associated with cybersecurity-related terms. The relevance and comprehensiveness of the strategy were corroborated through comparisons with previous reviews [23,24,25,26] and expanded to include IoT protocols, vehicular networks, and RF-based sensing systems. The complete search string and the grouping of descriptors are presented in Appendix A.

The document collection was conducted on 25 August 2025, using the Scopus and Web of Science databases. The selection of these databases aligns with previous studies that emphasize their complementarity and importance for systematic reviews [40,41]. In Scopus, 6284 records were retrieved, of which 4220 were excluded for not meeting the language and document-type criteria, including conference papers, book chapters, reviews, books, editorials, and publications in other languages. This reduction was also influenced by a temporary technical limitation in Scopus at that time, which restricted the maximum download to 5000 records per query. After the exclusions, 2163 scientific articles in English remained.

In the Web of Science database, 2514 records were initially retrieved; after excluding review articles, book chapters, editorial materials, early access papers, and retracted articles, 2415 documents remained. The combined set of articles from both databases was subsequently processed in RStudio using the remove.duplicated() function from the Bibliometrix package, resulting in 2364 unique scientific documents after deduplication. Next, a manual screening of metadata (from magazines without DOI, keywords, and affiliations) was performed, leading to the exclusion of 216 additional records and yielding a total of 2148 valid articles for the LLM-assisted semantic screening.

The review protocol was registered in the Open Science Framework (OSF) prior to the submission of this manuscript. The full protocol, the minimal documented triage script, and the final deduplicated dataset are publicly available at the following link: OSF Registration ID: https://osf.io/bqx7n/ (accessed on 11 December 2025).

### 2.2. Selection and Screening

**Figure 2:**
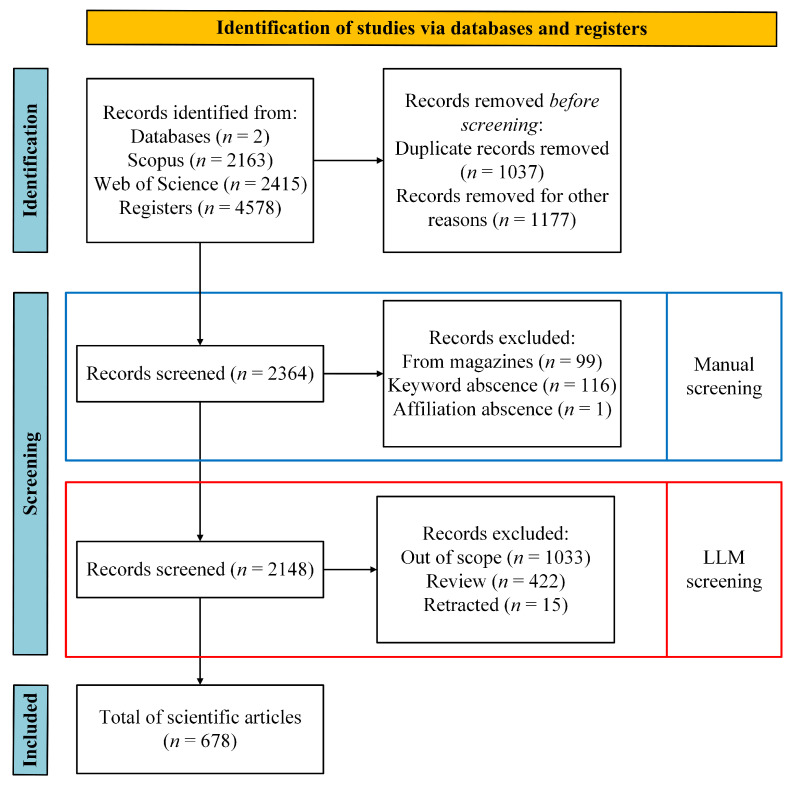
Adapted PRISMA 2020 flow diagram, highlighting the LLM-assisted screening stage.

Following the PRISMA 2020 protocol, the 2148 articles were subjected to two stages: (i) manual screening of titles, abstracts, and keywords according to the inclusion and exclusion criteria, and (ii) AI-assisted semantic screening (Python + OpenAI API), employed to confirm thematic relevance and reduce false positives. Figure 2 presents the adapted PRISMA flow diagram, highlighting the automated screening stage.

Figure 2 illustrates the inclusion of the LLM-assisted screening (highlighted in red), showing the number of automatic exclusions and subsequent manual reviews. The automated process also identified and removed records incorrectly classified as “Articles” under the DT tag, which in fact corresponded to reviews, as well as detected and eliminated retracted papers. This process comes after the manual screening procedure (highlighted in blue), which is an intrinsic step in PRISMA 2020 protocol.

After executing the automated process, 1462 articles were classified as out of scope, leaving 582 directly relevant and 104 labeled as “maybe”. The latter were manually re-evaluated, resulting in the inclusion of 96 articles that demonstrated a direct relationship between cybersecurity and radio frequency. The final corpus comprises 678 articles (7.7% of the initial total), published between 2009 and 2025.

It is important to clarify that the inputs analyzed in this study do not correspond to raw RF sensing data or physical signal measurements. Instead, the analysis relies on structured evidence extracted from peer-reviewed publications, including reported attack types, affected RF technologies, and application or experimental contexts described by the original authors. Consequently, signal-level characteristics such as sampling rates or noise distributions are not directly applicable. The representativeness of the analysis is therefore grounded in the diversity and scale of the scientific corpus, which spans multiple RF technologies and application domains, enabling the identification of systemic trends, research gaps, and recurring vulnerability patterns in RF cybersecurity.

In contrast to conventional systematic reviews that rely predominantly on keyword-based filtering and fully manual screening, the methodology adopted in this study incorporates an AI-assisted semantic screening stage. This design choice is particularly relevant for RF cybersecurity research, where heterogeneous terminology across technologies, attack types, and application domains can limit the effectiveness of purely lexical approaches [30,32]. By prioritizing semantic relevance while remaining aligned with the PRISMA 2020 framework, the proposed method supports improved scalability and consistency in large-scale literature screening.

### 2.3. LLM-Assisted Semantic Screening and Decision Rules

The semantic screening was performed using the Python RF Cybersecurity script, which integrates context retrieval (Retrieval-Augmented Generation—RAG) and contextual classification based on an LLM [35]. The process was executed locally using the official OpenAI API with the GPT-4o-mini model, without any training or fine-tuning stages.

**Figure 3:**
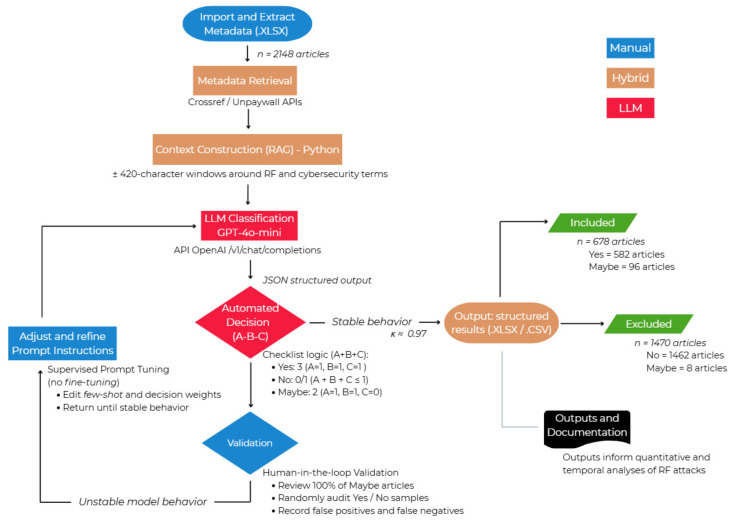
Flowchart of the LLM-assisted semantic screening and human-in-the-loop validation process. The workflow integrates RAG-based context construction, *A*–*B*–*C* checklist classification, supervised iterative prompt adjustment, and reliability assessment (κ≈0.97).

Figure 3 presents the flowchart of the automated LLM-based semantic screening process and the human-in-the-loop validation stage.

The execution flow comprised: (i) extraction of article metadata (title, abstract, and keywords) from the exported spreadsheets; (ii) data enrichment via the Crossref and Unpaywall APIs; (iii) context construction using ±420-character windows around terms related to RF and cybersecurity; and (iv) querying the Chat Completions API (/v1/chat/completions), employing prompt engineering based on the *A*–*B*–*C* checklist.

During this stage, the model simultaneously performed semantic classification and structured extraction of thematic evidence, populating fields such as RF_Technology, Protocol_Frequency, RF_Attack_Threat_Vulnerability, Application_Example, as well as IoT_Devices. These data were recorded in spreadsheets (.XLSX, .CSV) and served as the basis for the analyses presented in Section 4.1, Section 4.2 and Section 4.3.

The GPT-4o model, as described by [32], is an LLM based on a transformer architecture capable of estimating conditional probabilities of token sequences given a context. It is a model that is pretrained on large volumes of textual data that, in this study, was guided through prompt engineering, explicitly incorporating the defined inclusion and exclusion criteria [42,43,44]. This configuration enabled the model to operate as a contextualized classifier, maintaining decision traceability based on the full content of the articles. In the present study, the model operated under a few-shot learning regime without any additional retraining. Controlled (temperature=0.0, max_tokens, stop_token) were adopted to ensure more stable and auditable outputs (minimizing randomness across executions).

The adoption of the Chat Completions API, replacing the legacy Completions API, enabled structuring the interaction in a message-based format (system and user), allowing for more robust contextual instructions and greater output control. The responses were returned in JSON format, containing fields such as Cybersecurity_RF, RF_Technology, RF_Attack_Threat_Vulnerability, Evidence_Type, Confidence, Checklist_RF, as well as Checklist_Security. This arrangement standardizes the result structure and ensures traceability and reproducibility (field mapping and version control of the .XLSX and .CSV spreadsheets).

The inclusion and exclusion criteria were previously defined and applied uniformly throughout the entire screening process to ensure objectivity, consistency, and traceability of the results [32,45].

Four central axes were considered to determine the relevance of the articles to the analytical corpus: (i) evidence of RF technology: explicit mention of wireless communication protocols or technologies such as ZigBee, Bluetooth Low Energy (BLE), Wi-Fi, LoRa/LoRaWAN, RFID/NFC, GNSS, LTE/5G/6G, ISM band, UWB, V2X, and satellite systems; (ii) evidence of cybersecurity: presence of cyberattacks, threats, or defense mechanisms such as jamming, spoofing, replay, relay, eavesdropping, man-in-the-middle, and injection; (iii) direct link between RF and cybersecurity: the vulnerability or attack occurs at the physical communication channel, directly exploiting the radio frequency spectrum; and (iv) practical or experimental approach: use of testbeds, simulations, SDR devices (HackRF, USRP, RTL-SDR), or software tools such as GNU Radio.

Studies focused solely on performance (QoS), energy efficiency, antennas, propagation, or energy harvesting were excluded, as well as works addressing logical security (encryption/authentication) without relation to the RF channel. Articles dealing with purely non-RF protocols (such as standalone TCP/IP) and studies of side-channel attacks based on passive measurements that do not exploit the wireless communication medium were also removed.

The inclusion, exclusion, or review decision was automated through a binary A–B–C heuristic: (*A*) presence of evidence of RF technology; (*B*) presence of evidence of cybersecurity; (*C*) direct link between both. For each article, the LLM assigned binary scores (1 = present, 0 = absent), generating a score equals (A+B+C). The decision rules were as follows: score 3 (A=B=C=1) for YES; score 2 (A=1,B=1,C=0) for MAYBE; and score 1 or 0 (A+B+C≤1) for NO.

All “MAYBE” articles were manually reviewed under the human-in-the-loop approach, which also included random samples from the “YES” and “NO” categories. This procedure ensured the accuracy of the classifications and enabled the iterative refinement of the prompt until stable behavior consistent with the study’s methodological guidelines was achieved [35,38,46].

### 2.4. Reliability, Validation, and Prompt Refinement

The human-in-the-loop validation was conducted at two levels: (i) full review of the articles classified as “MAYBE” (n=104); and (ii) random verification of “YES” (n=150) and “NO” (n=300) samples. This dual-check procedure made it possible to assess the stability of the classifications and to reduce false positives and false negatives. A false positive was defined as any article initially classified as “YES” by the model but, after manual analysis, found to have no effective connection between cybersecurity and radio frequency. Similarly, false negatives corresponded to articles classified as “NO” by the LLM but later validated as relevant.

These observations align with recent evidence on the use of LLMs in systematic reviews, which report high levels of accuracy, although they still rely on human supervision for bias mitigation and quality control [47,48]. In the present study, semantic screening was implemented in Python, integrating RAG and contextual classification based on an LLM. The model analyzed metadata, excerpts retrieved from external databases, and, when available, the full text of the articles, thereby enhancing methodological traceability and reproducibility.

The agreement between the LLM and the human review was measured using Cohen’s kappa coefficient (κ) [49], which is commonly applied in systematic screenings involving LLMs [30,33,38,50]. The coefficient is defined as:

(1) $$\kappa =\frac{p_{o}-p_{e}}{1-p_{e}}$$

where:

- $p_{o}$ represents the proportion of observed agreement between the model and the human review;
- $p_{e}$ corresponds to the agreement expected by chance.

In this study, the observed values were $p_{o}=0.986$, $p_{e}=0.506$ e κ=0.97, indicating an almost perfect agreement according to the criteria of [49]. The procedures used to derive the observed agreement ($p_{o}$) and the expected agreement by chance ($p_{e}$), including the class distributions and validation samples underlying these values, are detailed in Appendix C. Similar results were reported by [38,50], who also applied the kappa coefficient in validations of LLM-assisted classifications, achieving substantial levels of agreement between human and automated evaluators.

During the development of the semantic screening stage, multiple versions of the prompt were tested, varying in the formulation of instructions, the structure of the A–B–C checklist, and the number of few-shot examples. Each version was applied to sample subsets (Sample, *n*) of the corpus, allowing for comparison between the performance of automated classifications and human decisions. Table 1 presents the evolution of the prompt versions and the corresponding observed (Agreement, %) and adjusted (κ) agreement indices between the LLM and human validation. The samples ranged from 200 to 554 articles, balanced among the “YES,” “MAYBE,” and “NO” categories, ensuring statistical representativeness and consistency in the comparative evaluation.

The progressive refinement of the prompt led to a substantial increase in agreement with human decisions, reaching κ≈0.97 in the final version. This result demonstrates stable and semantically consistent model behavior, showing that the supervised calibration process (human-in-the-loop) was effective at reducing ambiguities and aligning the automatic inferences with the methodological criteria of the study.

By combining classical scientometric principles with artificial intelligence, this study demonstrates the potential of LLM-assisted approaches to make systematic reviews more accurate, transparent, and reproducible [38,51,52].

## 3. Scientometric Analysis Results

### 3.1. General Information of the Corpus

Table 2 presents the general information of the bibliometric analysis, providing a comprehensive overview of the temporal evolution, productivity, and scientific collaboration in the field of cybersecurity applied to radio frequency technologies. The period considered covers publications from 2009 to 2025, totaling 678 documents distributed across 234 scientific sources. These numbers indicate that the topic has consolidated in recent years, following the expansion of the Internet of Things ecosystem [25,26,53] and the growing concerns about the security of connected devices [7,27,54,55,56,57,58].

The average annual growth rate of 28.01% highlights the rapid rise and recent relevance of the field in the international literature. The set of 1790 authors and the average of 4.12 co-authors per article reflect a collaborative and multidisciplinary pattern typical of consolidating research areas. Furthermore, the international co-authorship rate of 16.22% indicates the expansion of research networks and the strengthening of global partnerships among universities and specialized centers.

In summary, the data presented in Table 2 show that RF cybersecurity has emerged as a rapidly expanding scientific domain, characterized by thematic diversity, growing academic impact, and strong international cooperation. This initial overview establishes the foundation for the subsequent analyses on productivity, collaboration networks, and thematic trends.

### 3.2. Evolution of Scientific Production

**Figure 4:**
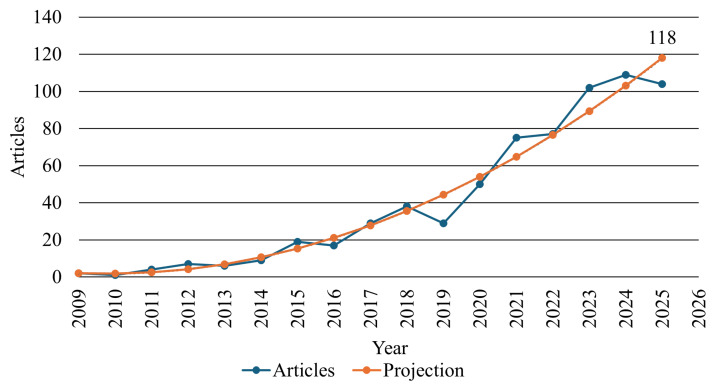
Annual scientific production.

Figure 4 shows, in blue, the annual evolution of scientific production on cybersecurity in radio frequency technologies between 2009 and 2025. The number of publications has grown continuously, with a particularly pronounced acceleration after 2018, when the topic began to receive greater attention from the scientific community. This trend is commonly associated with the increasing number of IoT devices and the consequent expansion of attack surfaces in connected systems [6,58,59,60,61,62,63].

The marked increase in publications observed after 2018 can be attributed to a set of converging technological and contextual factors rather than to the initial emergence of IoT or smart city concepts. While these paradigms predate this period, the years following 2018 correspond to their large-scale operational deployment, higher device density, and growing dependence on RF-based communications in critical urban and industrial systems [5,6]. In particular, the widespread adoption of LPWAN technologies operating in unlicensed spectrum, together with the commercial rollout of 5G networks, introduced new scalability, latency, and connectivity paradigms, significantly expanding the RF attack surface [11]. These developments help explain the sustained growth in scientific output related to RF cybersecurity observed in Figure 4.

This evolution reflects a growing research focus on the security of wireless networks, RF protocols, and critical devices, with particular emphasis on applications in smart cities, critical infrastructures, and industrial systems [7,64,65,66,67,68,69]. The observed growth pattern further suggests the consolidation of RF cybersecurity as an emerging interdisciplinary core that bridges electrical engineering, computer science, and applied cybersecurity [58,70,71,72,73].

Also in Figure 4, the orange curve represents the projection of future production, obtained using a second-degree polynomial regressor with parameters $a_{0}=2.027\times 10^{6}$, $a_{1}=2018.096$, and $a_{2}=0.502$, resulting in $R^{2}=0.967$. The model estimates that the number of publications on the topic will reach approximately 118 in 2025 and 134 in 2026, indicating the continuation of the growth trend and the consolidation of this domain as an area of scientific and technological opportunity.

Table 3 complements this analysis by presenting the evolution of the average number of citations per article over the same period, highlighting the temporal impact of academic production. The earliest works show higher average citation counts, reflecting their seminal role in establishing the theoretical foundations of the field. Starting in 2018, the sharp increase in publication volume is accompanied by a slight reduction in the average number of citations, an expected behavior in rapidly expanding areas, where more recent studies have not yet achieved wide dissemination. These results reinforce the transition from an initial phase of conceptual consolidation to a stage of scientific maturity, characterized by the diversification of approaches and the strengthening of the field’s international recognition.

**Figure 5:**
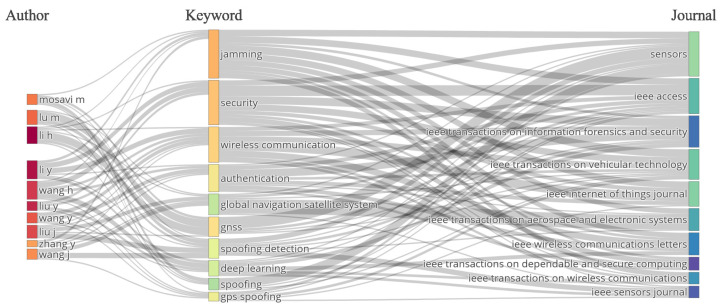
Three Field Plot which associates article authors, most used keywords and journals.

Figure 5 presents the Three-Field Plot diagram [74], which relates the main article authors (AU), keywords (DE), and publication sources (SO), highlighting the ten most representative elements of the scientific production on RF cybersecurity. This visualization makes it possible to identify how the core research topics are distributed among the most productive authors and the highest-impact journals. Notable contributors include Mosavi M., Lu M., Li H., Li Y., and Wang H., who are strongly associated with terms such as “jamming,” “security,” “wireless communication,” “authentication,” and “GNSS/GPS spoofing” [75,76,77,78,79]. This thematic concentration indicates the predominance of studies focused on the analysis of attacks and the development of defense mechanisms in wireless communication systems, particularly within the physical and data link layers of the OSI model [80].

Among the most relevant journals, Sensors, IEEE Access, IEEE Transactions on Information Forensics and Security, IEEE Transactions on Vehicular Technology, and the IEEE Internet of Things Journal emerge as the primary dissemination channels for RF cybersecurity research. The analysis reveals three main axes of convergence: (i) a research community centered on jamming and spoofing analysis and mitigation [54,81,82,83,84]; (ii) the growing incorporation of machine learning and intrusion detection techniques [85,86,87,88]; and (iii) the concentration of contributions in journals dedicated to the reliability and protection of IoT and wireless systems [7,11,57,89]. Together, these patterns illustrate the progressive maturation of RF cybersecurity as a research field aligned with contemporary challenges in wireless communication security.

### 3.3. Most Relevant Sources

Table 4 presents the most relevant journals in the scientific production on cybersecurity in RF technologies. Most studies are concentrated in high-impact journals in the fields of engineering, telecommunications, and information technology. MDPI Sensors has the highest number of publications (41 articles), followed by the IEEE Internet of Things Journal (34), IEEE Transactions on Vehicular Technology (32), and IEEE Access (31). This concentration reflects the growing interest in research focused on the security of IoT devices, wireless communication, and embedded systems.

The predominance of IEEE journals reinforces the technical and applied profile of the field, highlighting the emphasis on detection methods, authentication, attack mitigation, and the protection of RF communication protocols [57,90,91]. In parallel, the prominent presence of Sensors, a multidisciplinary open access journal, highlights the relevance of research that integrates hardware, sensor networks, and the security of connected devices [92,93,94]. Together, these publication venues illustrate the consolidation of RF cybersecurity as an interdisciplinary domain at the intersection of engineering, computing, and information security [71,72,73].

**Figure 6:**
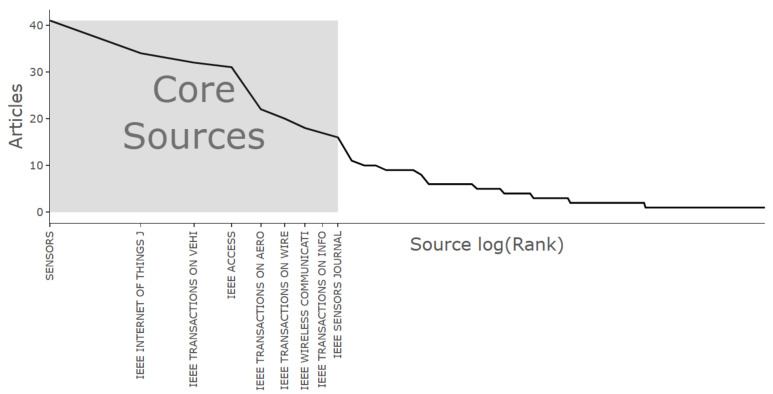
Bradford’s Law.

Figure 6 presents the chart based on Bradford’s Law [95], which illustrates the distribution of publications across journals and identifies the core group of the most productive sources. MDPI Sensors occupies the central region (Core Sources), standing out in relevance compared to other journals, thereby confirming its role as the primary dissemination channel in the field.

**Figure 7:**
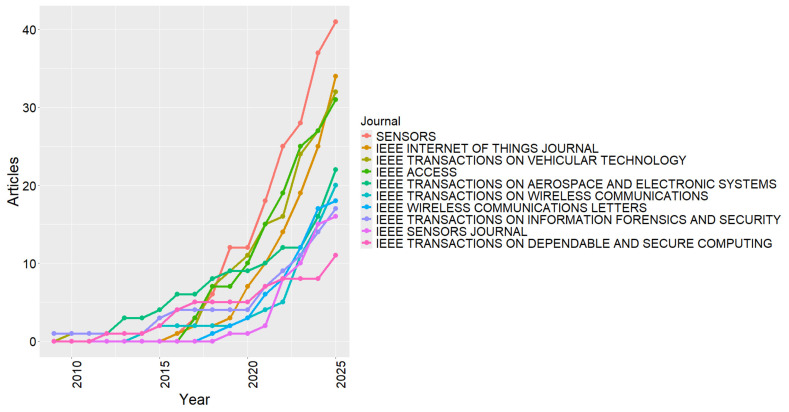
Sources’ production over time.

Figure 7 shows the temporal evolution of the main publication sources, illustrating how the outlets disseminating RF cybersecurity research have diversified over time. This analysis is relevant to reveal shifts in editorial focus and to contextualize how the field has expanded beyond a small set of specialized venues. In the early years, publications were largely concentrated in a limited number of journals. From 2016 onward, an increased participation of outlets such as IEEE Transactions on Vehicular Technology and IEEE Transactions on Aerospace and Electronic Systems can be observed, reflecting the growing integration of RF cybersecurity topics with embedded systems, vehicular communications, and safety-critical wireless applications [96,97].

The most significant growth occurs from 2018 onward, driven by the expansion of publications in MDPI Sensors, IEEE Internet of Things Journal, and IEEE Access, which began to lead the dissemination of studies on IoT, wireless networks, and the protection of connected devices [7,11,89]. This movement reflects both technological advancement and the popularization of open access, high-impact journals, which facilitate the global dissemination of research findings. Thus, the observed trend confirms the consolidation and diversification of publication sources, reinforcing that RF cybersecurity has established itself as a stable, interdisciplinary field in continuous international expansion.

### 3.4. Productivity and Impact by Authors

Table 5 presents the most productive article authors in the field of RF cybersecurity. Li H. and Li Y. stand out with 17 publications each, followed by Liu Y. and Wang H., both with 16 articles. These researchers have consistently contributed to advancing knowledge, particularly in topics related to jamming and spoofing attacks, device authentication, and security in wireless technologies [98,99,100,101].

The fractional production values, ranging from 2.51 to 4.95, reflect different levels of collaboration among authors and research groups. Researchers such as Mosavi M. and Wang Y. exhibit higher individual representativeness, suggesting leadership roles in major projects and reference publications [75,90,102]. Overall, the group of the most productive authors represents a consolidated and highly cooperative scientific community, predominantly composed of Asian researchers who have driven the international advancement of studies on cybersecurity in radio frequency communications.

**Figure 8:**
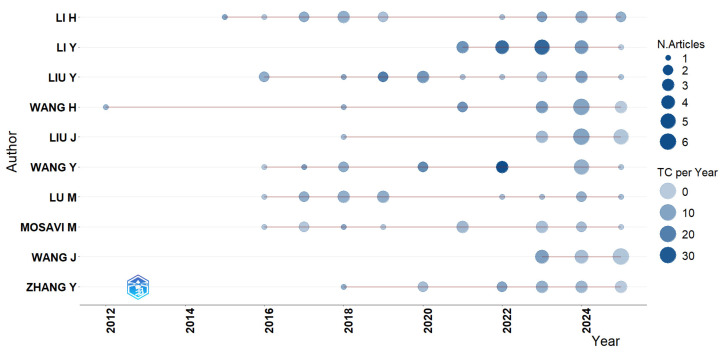
Article authors’ (AU) production over time.

Figure 8 complements this analysis by illustrating the temporal evolution of productivity and impact among the main authors. Each bubble represents the number of publications per year; the larger the diameter, the greater the volume of articles, and darker shades indicate a higher average number of annual citations (TC per Year), reflecting the scientific impact of their contributions.

Authors such as Li H., Li Y., Liu Y., and Wang H. have maintained a stable and productive trajectory throughout the analyzed period, combining a high number of publications with strong citation impact, factors that reinforce their influence in the theoretical consolidation of the field. In more recent years, Mosavi M., Lu M., and Zhang Y. have stood out by expanding their contributions and introducing approaches based on machine learning, intelligent networks, and critical systems security [61,75,79]. This dynamic highlights the transition from a community centered on pioneering researchers to an expanding collaborative ecosystem, in which different generations of authors converge around emerging themes in RF cybersecurity.

**Figure 9:**
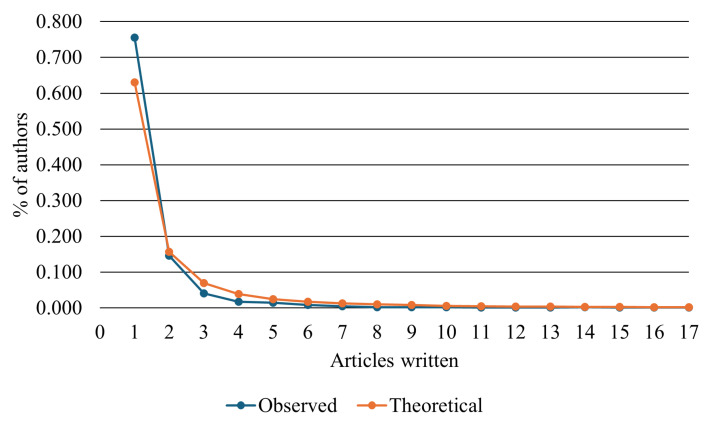
Authors’ productivity according to Lotka’s law.

Figure 9 shows the distribution of author productivity according to Lotka’s Law [103], which relates the proportion of researchers to the number of publications. The chart compares the theoretical curve (inverse square law) with the empirical results observed in this study, indicating the degree of adherence between them.

The observed curve (in blue) shows good correspondence with the theoretical curve (in orange), suggesting that a small fraction of authors, less than 0.1%, accounts for most of the publications, while the majority contribute only sporadically. This pattern is characteristic of consolidating scientific fields, in which production tends to concentrate within leading research groups.

In the context of RF cybersecurity, such concentration reflects the role of a limited number of research groups in shaping core research problems and methodological approaches, while also highlighting the importance of collaboration networks for the maturation and diversification of the field.

### 3.5. Scientific Productivity by Institutions

Table 6 presents the institutions with the highest publication volumes in the field of radio frequency cybersecurity. A strong predominance of Chinese universities is observed, with Xidian University (24 articles) and Tsinghua University (22 articles) standing out, followed by Beijing Jiaotong University and Southeast University, both with 21 publications. These universities have established themselves as centers of advanced research in wireless communications, embedded systems, and IoT network security, playing a central role in the scientific and technological advancement of the field [104,105,106,107].

Institutions such as Nanjing University of Posts and Telecommunications, Beihang University, and Xi’an Jiaotong University show strong engagement in secure communication protocols and attack mitigation in RF networks [108,109]. Outside the continental Chinese context, Hong Kong Polytechnic University and Nanyang Technological University (Singapore) stand out for strengthening regional cooperation and expanding the integration of Asian research centers into the global landscape. Overall, the institutional distribution confirms the geographical concentration of scientific excellence in Asia, particularly in China, which leads the development of innovative solutions for the security of wireless communication systems.

**Figure 10:**
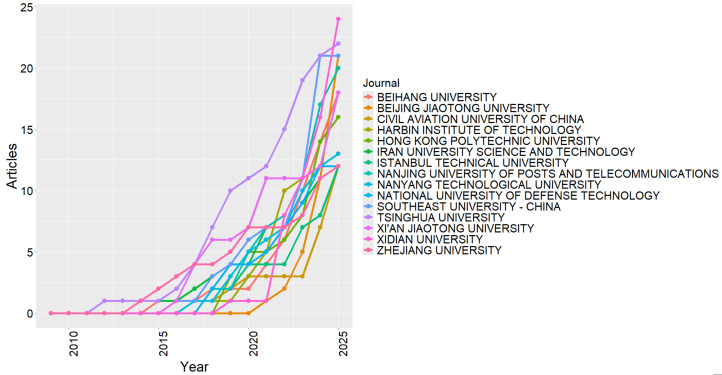
Affiliations’ production over time.

Figure 10 complements this analysis by illustrating the temporal evolution of productivity among the main universities active in the field. A consistent growth trend is observed starting in 2015, driven by the increasing number of publications from institutions already ranked among the most productive, such as Tsinghua University, Beijing Jiaotong University, and Xidian University. This movement parallels the intensification of research focused on IoT network protection and wireless communications, consolidating the role of these universities as international reference centers.

Starting in 2019, a more diversified expansion can be observed, marked by the entry of new institutions and the strengthening of international collaborations, particularly with universities in Hong Kong, Singapore, and European countries. This expansion indicates a movement toward internationalization and scientific cooperation, in which the topic is no longer concentrated in a few Asian hubs but has become part of a global research network on cybersecurity in RF systems. The temporal pattern, therefore, reveals a field in full consolidation, sustained by continuous growth, interinstitutional collaboration, and the progressive increase of international visibility.

### 3.6. Scientific Productivity by Country

**Figure 11:**
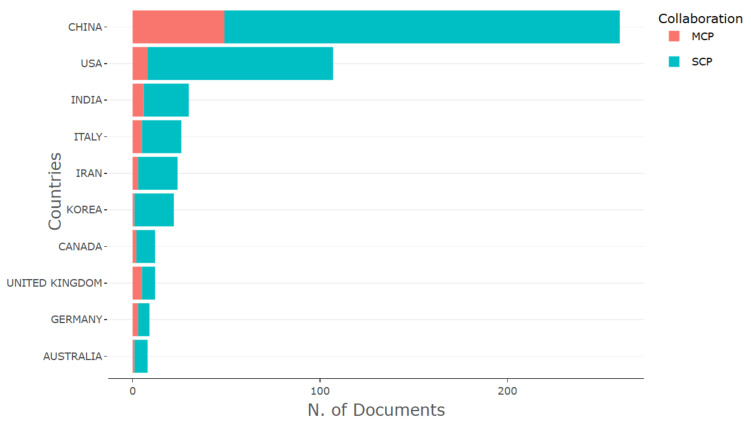
Author Countries.

Figure 11 shows the distribution of scientific production by country, distinguishing publications with exclusively national authorship (SCP—Single Country Publications) from those resulting from international collaborations (MCP—Multiple Country Publications).

It is observed that most countries maintain a predominance of publications with domestic authorship (SCP), reflecting the strengthening of consolidated national research groups. Conversely, nations with higher rates of international collaboration, measured by MCP, tend to exhibit greater scientific impact, corroborating the pattern already evidenced by the most productive affiliations and authors (Table 6, Figure 8 and Figure 10). Countries such as South Korea and Australia still display limited participation in multilateral networks, indicating potential for expansion toward future international collaborations.

**Figure 12:**
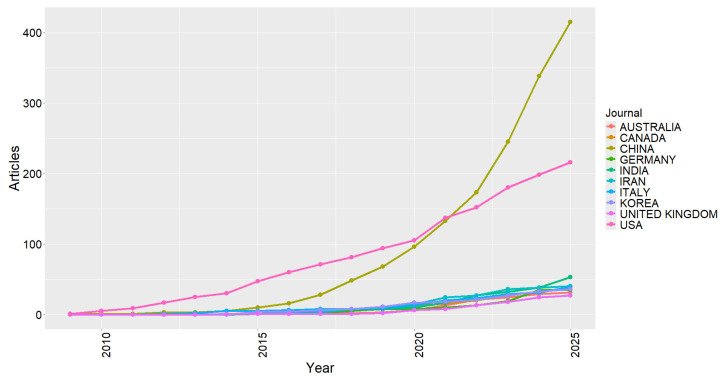
Countries’ production over time.

Figure 12 shows the temporal evolution of scientific production by country, highlighting the significant growth of China throughout the analyzed period. The continuous increase in publications since 2015 confirms the country’s central role in consolidating research on cybersecurity applied to RF systems, driven by national policies on technological innovation and information security. In addition to quantitative growth, there is a progressive diversification of global scientific output, with increasing participation from countries such as the United States, India, France, and Italy, which have intensified their contributions in recent years.

In recent years, scientific production has come to reflect a more distributed collaboration landscape, marked by the inclusion of new Asian countries and the strengthening of European research networks. This internationalization movement indicates the transition from a field once concentrated in a few hubs to a more diversified and cooperative global ecosystem. Different regions of the world now contribute to the development of solutions aimed at protecting critical infrastructures and wireless communications. The observed pattern reinforces the consolidation of RF cybersecurity as a mature research area of strategic international relevance.

### 3.7. Countries with the Highest Global Citation Impact

Table 7 presents the countries with the highest total number of citations in publications on cybersecurity in radio frequency technologies. China ranks first with 4154 citations, followed by the United States with 3286 citations, demonstrating the leading role of these two nations in the consolidation and dissemination of scientific knowledge on the subject. This result reflects both the substantial volume of scientific output and the growing strategic relevance of RF cybersecurity research in these countries.

Next, Italy, Iran, France, Canada, India, the United Kingdom, South Korea, and Australia stand out, completing the group of the ten most-cited countries. These results demonstrate the presence of established research hubs across Europe, North America, and Asia, with an emphasis on applications related to the Internet of Things, wireless networks, and critical systems. Overall, the geographical distribution of citations indicates a globally active and collaborative research field, characterized by Asian leadership in publication volume and strong Western participation in the consolidation of research on cybersecurity applied to radio frequency.

The metric “Average Article Citations”, presented in Table 7, represents the average number of citations per article and helps in the relative interpretation of scientific impact. However, it should not be analyzed in isolation, as it may vary depending on the total number of publications from each country and the maturity of national research programs addressing RF security in critical and large-scale systems.

Table 8 presents the ten most globally cited articles in the field of cybersecurity in RF technologies. The results show that the highest-impact works are concentrated on attacks and countermeasures in wireless communication systems, addressing topics such as spoofing, jamming, device authentication, cognitive communications, and IoT network security, particularly in contexts where RF vulnerabilities directly affect sensing, positioning, synchronization, and control functions. The study by [110], published in the International Journal of Critical Infrastructure Protection, has the highest number of citations and stands out as one of the pioneering works on vulnerabilities in positioning systems and critical infrastructure. Following that, the article by [111], published in IEEE Wireless Communications, shows high annual impact and reflects the growing interest in cooperative security and machine learning approaches applied to RF network protection.

Also noteworthy is the work by [112], published in IEEE Transactions on Cognitive Communications and Networking, which presents the highest normalized citation index (6.68), demonstrating its recent influence and relevance to the advancement of cognitive communication techniques and adaptive attack mitigation. Other studies, such as those by [113,114], further reinforce the importance of research on security and integrity in GNSS systems and spoofing detection, illustrating the field’s transition from conceptual studies to practical solutions with high scientific impact.

### 3.8. Dynamics and Frequency of Keywords

**Figure 13:**
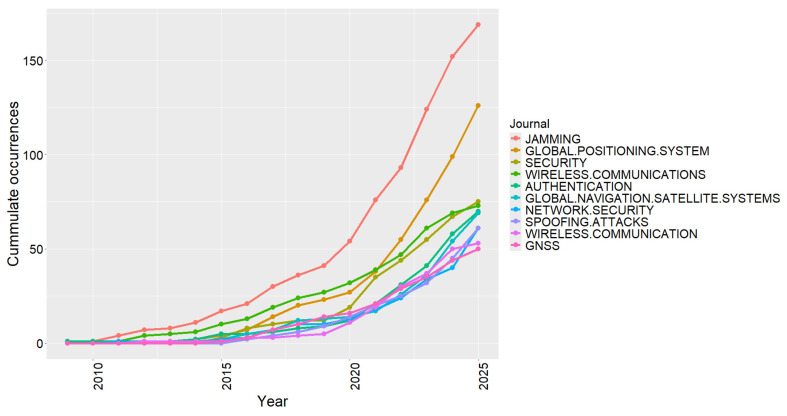
Words’ frequency over time.

Figure 13 shows the temporal evolution of the most recurrent keywords in the literature on cybersecurity in radio frequency technologies, highlighting the gradual transformation of the field’s main thematic axes. In the early stages (2009–2014), the terms “jamming”, “authentication”, and “wireless communications” stand out, reflecting the predominant focus on denial-of-service attacks and fundamental authentication strategies in wireless systems. This initial phase consolidated the conceptual and methodological foundations of the area, establishing the theoretical basis for subsequent studies on vulnerabilities and risk mitigation in communication protocols.

From 2016 onward, the scientific vocabulary became more diversified, with the emergence of terms such as “spoofing attacks”, “network security”, and “GNSS”, indicating the convergence between satellite positioning, network security, and critical communications. Between 2019 and 2023, the higher frequency of expressions such as “global positioning system” and “wireless communications” demonstrates the maturation of the field and the growing interest in smart infrastructures, IoT devices, and the protection of heterogeneous networks, where RF signals play a dual role as communication and sensing enablers. Despite this thematic expansion, the term “jamming” remained constant, reaffirming its role as a central and persistent concept in research on attacks and defenses in RF systems. Taken together, these results demonstrate the transition from an initial technical focus to an interdisciplinary paradigm in which attack detection, cryptography, and defense have become consolidated as the core pillars of RF cybersecurity research.

**Figure 14:**
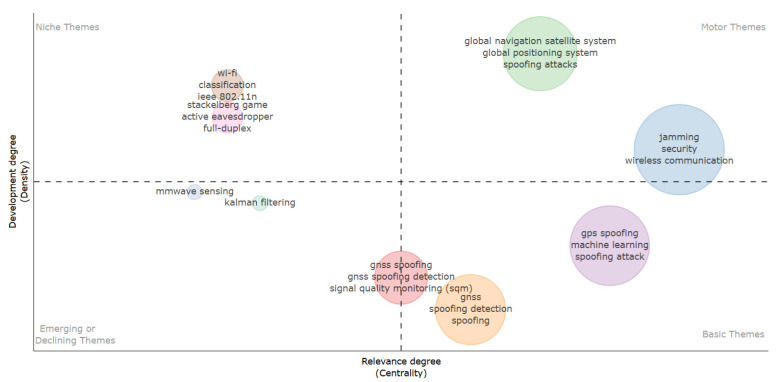
Thematic Map.

Figure 14 complements this analysis by presenting a thematic map of the research front, divided into four quadrants according to the degree of relevance (centrality) and development (density) of the themes. Each cluster results from the relationship among co-occurring keywords, allowing the identification of the conceptual axes that support the structure of the field. The motor themes quadrant brings together the most influential and emerging topics, indicating the areas with the greatest potential for technological innovation and future impact [119], particularly in applications where RF resilience directly affects sensing accuracy, availability, and system trustworthiness.

In the Motor Themes quadrant (upper right), terms such as “jamming”, “security”, “wireless communication”, and “GNSS” appear with high centrality and density, forming the core pillars of RF cybersecurity. Niche Themes (upper left), including “Wi-Fi” and “full-duplex”, are well-developed but less connected to the main research flows. Basic Themes (lower right), like “machine learning” and “GPS spoofing”, show high relevance but are still under methodological development. Meanwhile, the Emerging or Declining Themes (lower left), such as “Kalman filtering” and “mmWave sensing”, may signal either nascent investigations or reduced scientific attention. This thematic layout offers a structured view of the maturity and evolution of key topics.

The map was constructed using co-occurrence analysis with centrality–density clustering, revealing both the stable conceptual foundations and the emerging directions in the field. The results illustrate the scientific maturity and increasing global relevance of RF cybersecurity, particularly in response to the growing demands of secure and resilient wireless communication systems.

### 3.9. International Scientific Collaboration

**Figure 15:**
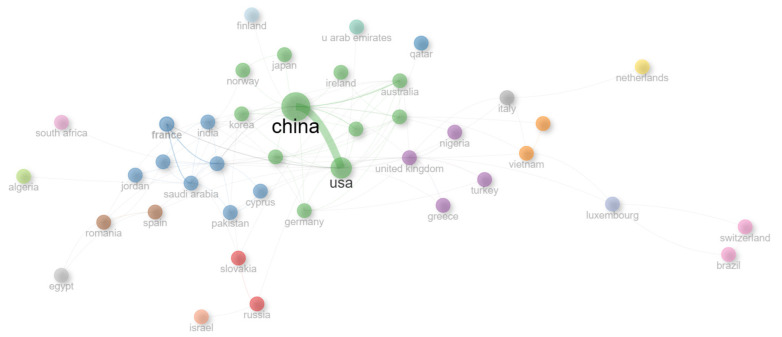
International collaboration network among countries in RF cybersecurity research. Nodes represent countries, and edges indicate co-authorship relationships.

Figure 15 presents the global network of scientific collaboration in the field of cybersecurity in RF technologies, highlighting the connections among the main knowledge-producing countries. The clusters are identified by the node colors. The collaboration strength between countries is denoted by the edge thickness. China and the United States form the central and most densely connected core of the network (green cluster), sustaining the most significant international partnership in terms of co-publications and researcher exchange. This bilateral connection reflects the scientific and technological leadership of these nations, which combine high productivity with continuous investment in applied research on IoT network security, wireless communications, and GNSS systems.

The second most colaborative cluster is the blue cluster which contains France, India, Pakistan and others. India has a closer collaboration with South Korea that is presented in the green cluster. Around this axis, complementary collaborations stand out with countries such as Germany, Australia, the United Kingdom, France, and India, which act as intermediate nodes in the dissemination and diversification of scientific partnerships. These countries often contribute specialized expertise in areas such as vehicular communications, satellite systems, industrial IoT, and security evaluation frameworks, reinforcing the multidimensional nature of RF cybersecurity research. The map also reveals the emergence of new regional hubs, particularly Saudi Arabia, South Korea, and Singapore, which expand Asian representation in the global landscape. Conversely, participation from Latin American and African countries remains incipient, with occasional cooperation links involving Brazil, South Africa, and Egypt, indicating potential for expansion and strengthening of intercontinental collaboration networks.

Overall, the network structure reveals a collaborative model that remains concentrated but is undergoing expansion, with China playing a central articulating role by connecting research centers across Asia, Europe, and North America. This configuration confirms that RF cybersecurity has consolidated as a globalized and cooperative field, in which the integration between leading and emerging countries is essential for developing innovative solutions and addressing cybersecurity challenges on an international scale.

## 4. Trends, Vulnerabilities, and Technological Impact

### 4.1. Temporal Trends in RF Cyberattacks

**Figure 16:**
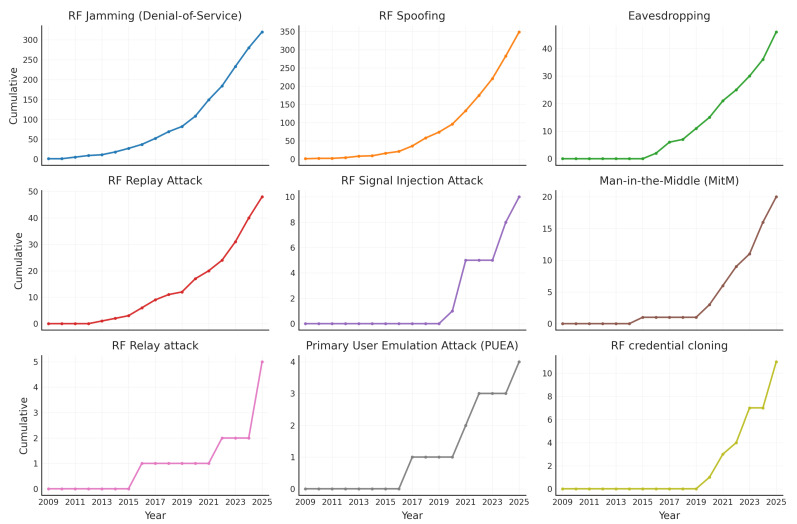
Cumulative evolution of the main cyberattacks in radio frequency technologies reported in scientific articles between 2009 and 2025. Each subplot represents a specific attack category, including jamming, spoofing, replay, and others, highlighting the cumulative growth of each type over the analyzed period.

Figure 16 illustrates the cumulative evolution of the main cyberattacks in radio frequency technologies reported in scientific articles published between 2009 and 2025. The use of multiple subplots allows comparison of the temporal behavior of each attack category, highlighting different growth rates and maturity levels across research lines. This visualization reveals a significant increase in the volume of studies focused on wireless communication security, with emphasis on jamming (denial-of-service) and spoofing attacks, while other vectors such as replay, relay, and credential cloning show more moderate growth over the analyzed period.

The jamming attack, characterized by the intentional emission of interference in the communication channel, has shown an upward trend since 2015, reflecting its persistence as one of the most studied threats at the physical layer of RF systems [70,120,121,122,123,124,125]. This sustained interest can be attributed to the low technical barrier for execution, the direct coupling with spectrum occupancy, and the high impact of jamming on availability-sensitive applications such as industrial control, vehicular systems, and IoT deployments operating in unlicensed bands.

Similarly, spoofing, which involves the falsification of signals or identities, has exhibited an even more pronounced increase since 2018, accompanying the expansion of GNSS, IoT, and critical communication technologies [118,126,127,128,129]. From a sensing and communication perspective, spoofing exploits inherent trust assumptions in RF receivers and protocol designs, particularly in systems where signal authenticity is inferred from physical-layer characteristics rather than cryptographic guarantees. This explains its prominence in GNSS-based applications, autonomous systems, and low-power IoT devices.

Other vectors, such as replay attacks, eavesdropping, signal injection, and man-in-the-middle, also show a gradual increase after 2020, a period during which the diversification of connected devices significantly expanded attack surfaces [11,77,130,131,132,133,134,135]. These attacks are often associated with higher-layer protocol interactions but depend on RF channel access and timing characteristics, making them increasingly relevant in heterogeneous IoT environments where sensing, communication, and control are tightly coupled.

Meanwhile, categories such as relay attack, Primary User Emulation Attack (PUEA), and RF credential cloning have emerged more recently, with modest curves that reflect research fronts still in consolidation [136,137,138,139,140]. Their slower growth reflects the fact that these attack vectors are typically investigated in technology- and context-specific scenarios, such as cognitive radio networks, RFID systems, or proximity-based authentication, rather than across broad classes of RF communication systems.

The use of a layout with multiple subplots prevents visual overlap and highlights the differences in pace and maturity among the categories. Overall, the results indicate that the field of RF cybersecurity is evolving from classical and extensively studied attacks, such as jamming and spoofing, toward a reconfiguration of known threats in new application contexts, in parallel with the growing complexity and interconnectivity of communication ecosystems.

### 4.2. Mapping RF to Cyberattack Types

**Figure 17:**
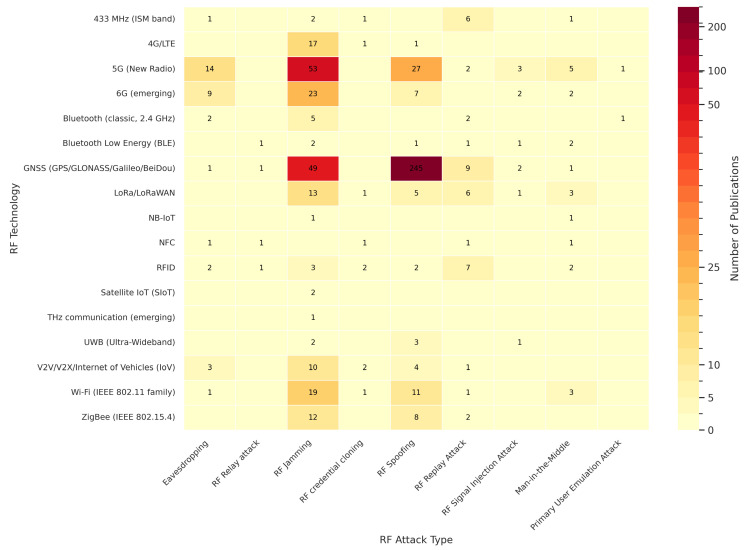
Heatmap illustrating the occurrence frequency of cyberattack types in radio frequency systems reported in the scientific literature, associating each attack category with the different communication technologies analyzed.

Figure 17 presents a heatmap correlating the main radio frequency communication technologies with the different types of cyberattacks identified in the literature. This visualization enables an integrated understanding of how vulnerabilities are distributed across protocols, frequency bands, and communication layers, revealing both consolidated patterns and emerging research gaps. Importantly, these patterns reflect differences in how each technology exposes the RF channel to interference, manipulation, or signal impersonation. The color intensity represents the number of publications related to each technology–attack combination, highlighting the most extensively studied areas as well as those that remain underexplored.

The concentration of studies on the Global Navigation Satellite System (GNSS/GPS/ GLONASS/Galileo/BeiDou) is particularly significant, especially for spoofing (245 publications) and jamming (49 publications). This prominence underscores the critical importance of positioning technologies in strategic applications such as autonomous transportation, defense, logistics, and network synchronization, where signal reliability is essential [96,110,141,142,143,144,145]. The recurrence of these attack vectors confirms that intentional GNSS signal manipulation remains one of the most exploited vulnerabilities in RF systems, largely due to the open and broadcast nature of satellite signals, which makes them inherently susceptible to interference and signal falsification at the RF level [104,128,129,146,147,148].

Fifth- and sixth-generation mobile communications (5G and 6G) also stand out due to the high incidence of studies on jamming, spoofing, and eavesdropping, reflecting growing concern over the robustness of next-generation wireless infrastructures [8,14,149,150,151,152]. The emphasis on these technologies in the literature is explained not only by their widespread adoption, but also by their reliance on complex RF features, such as dynamic spectrum access, beamforming, and dense cell deployments, which increase sensitivity to interference, spoofing, and passive interception at the physical layer [4,5,150,151,152].

In IoT and local communication technologies such as LoRa/LoRaWAN, ZigBee, Bluetooth, BLE, and Wi-Fi, jamming continues to be the most recurrent vulnerability, reflecting these networks’ susceptibility to deliberate interference and their limited spectral isolation mechanisms [89,91,153,154,155,156]. Recent studies also report replay, relay, and man-in-the-middle (MitM) attacks, associated with authentication flaws and the distributed or cooperative nature of these protocols [131,157,158,159,160]. Taken as a whole, while jamming remains dominant, IoT technologies exhibit a progressively diversified threat landscape, reflecting the combination of constrained devices, shared spectrum usage, and lightweight authentication mechanisms typical of these RF ecosystems [57,63,122,161,162,163].

In this context, Table 9 complements the previous discussion by summarizing the ten most frequent combinations of radio frequency technologies and cyberattack types reported in published articles. It can be observed that spoofing in GNSS systems ranks first, followed by jamming attacks in 5G, 6G, Wi-Fi, 4G/LTE, LoRa/LoRaWAN, and ZigBee networks. This pattern indicates that research efforts remain concentrated on well-established technologies, whereas emerging protocols are still comparatively underexplored and thus represent relevant directions for future investigation.

From this mapping, it becomes evident that vulnerabilities in RF systems are unevenly distributed across technologies. This uneven distribution reflects not only differences in protocol design, but also how each technology interacts with the RF medium, which has direct implications for risk prioritization and the definition of mitigation strategies.

### 4.3. Cybersecurity Implications for IoT and Wireless Systems

Table 10 organizes representative RF-based cyberattacks observed in IoT, highlighting the corresponding RF technologies, affected device categories, real-world application examples, and the key studies that document these vulnerabilities across different domains. This organization makes explicit how differences in RF communication characteristics, deployment scale, and device constraints translate into distinct security implications across IoT and wireless systems.

Many of the RF technologies addressed in this study, such as BLE, ZigBee, Wi-Fi, GNSS, LoRa/LoRaWAN, and RFID, are integral to modern IoT ecosystems and wireless infrastructures. Their prevalence in the literature is closely linked to their reliance on open or shared spectrum, low-power operation, and broadcast communication models, which expose the RF channel to interference, manipulation, and unauthorized observation.

The vulnerabilities observed across these systems, including spoofing, jamming, replay, relay, eavesdropping, and RF signal injection, have been consistently demonstrated in the literature through empirical experiments and large-scale evaluations. For example, works such as [6,154,155,167,169] show how jamming and spoofing can critically degrade navigation accuracy, disrupt wireless connectivity, and compromise device authentication in domains ranging from UAV control to smart-home automation.

The results summarized in Table 10 also reveal that certain RF technologies are more frequently associated with specific classes of vulnerabilities in the literature. For instance, jamming is prevalent in LoRa/LoRaWAN, Wi-Fi, and ZigBee systems [120,155], while GNSS technologies are especially sensitive to spoofing [67,186]. Replay attacks frequently target Bluetooth/BLE, ZigBee, and 433 MHz systems [162,191], and MitM attacks are recurrent in LoRa and RFID infrastructures [181,194]. These associations underscore the need for technology-specific countermeasures and highlight the heterogeneous nature of the RF threat landscape, in which each protocol faces distinct exposure patterns and security requirements within IoT and wireless environments. Such patterns emerge not from isolated weaknesses, but from how protocol design choices, spectrum usage, and device capabilities interact at the RF layer.

Taken together, these findings show that reports of RF vulnerabilities in the literature are not uniformly distributed across technologies, but instead follow protocol-specific and device-specific patterns. The range of affected systems, including UAVs, autonomous vehicles, smart locks, industrial nodes, and satellite-IoT components, illustrates that each communication technology faces distinct operational constraints and exposure conditions. By making these patterns explicit, the mapping helps identify where mitigation efforts should be prioritized and supports the design of protection strategies tailored to the characteristics and threat profiles of each protocol. This perspective is particularly relevant for designers, operators, and regulators of IoT and wireless systems, who must balance performance, scalability, and security under RF-constrained conditions.

## 5. Discussion

The analysis shows that cybersecurity research applied to radio frequency technologies has developed around two principal thematic axes. The first concerns signal integrity and authentication in GNSS and cellular networks, where spoofing and jamming remain dominant issues due to their impact on navigation, synchronization, and critical communication infrastructures [2,150,195,196,197,198,199,200]. The second axis relates to the resilience of IoT and short-range wireless systems, which face an increasingly diverse set of threats, such as replay, relay, eavesdropping, and man-in-the-middle attacks, driven by the expansion of connected devices and the distributed nature of modern wireless environments [6,54,58,61,63,67,69,71,72,153,155,164,185]. These two directions depict a research landscape that is progressively integrating RF security into discussions on spectral resilience, interoperability, and cyber–physical protection.

Beyond the overall growth in publication volume, the observed increase in co-authorship and international collaboration suggests a gradual consolidation of RF cybersecurity as a research domain. Collaborative research patterns are increasingly common in areas that involve heterogeneous technologies and complex system interactions, such as IoT and wireless communication security. In this context, the rising collaboration rate reflects the interdisciplinary nature of RF cybersecurity and the need to integrate expertise from communications, embedded systems, and security engineering.

From a temporal and geographical perspective, the scientometric evidence reveals sustained growth in RF cybersecurity publications between 2009 and 2025, accompanied by a concentration of research activity in China and the United States, alongside strong contributions from other Asian institutions. This international research structure has supported advances in the detection, mitigation, and modeling of RF-based threats, reinforcing the global relevance of the field.

The analysis of scientific influence reinforces this trajectory. Journals such as Sensors, the IEEE Internet of Things Journal, and several IEEE Transactions appear as recurring venues for RF security research. Citation and keyword analyses point to growing interest in artificial intelligence for RF anomaly detection, the protection of heterogeneous IoT environments, and the design of spectrally resilient wireless systems. These trends illustrate how RF cybersecurity research is increasingly aligned with broader technological developments related to autonomous mobility, critical infrastructure protection, and distributed sensing.

With respect to the threat landscape, the literature confirms the prominence of jamming and spoofing as the most extensively studied attack vectors, owing to their practicality and disruptive potential at the physical layer. At the same time, the increasing focus on replay, relay, credential cloning, and man-in-the-middle attacks reflects the expansion of the RF attack surface in ecosystems characterized by ubiquitous connectivity and resource-constrained devices. Technologies such as GNSS, 5G/6G, Wi-Fi, and LoRaWAN feature prominently in cybersecurity studies not only due to their widespread deployment, but also because vulnerabilities in these systems can propagate across interconnected communication layers.

From a practical standpoint, the technology- and attack-oriented mapping presented in this study can support more informed decision-making in the design and deployment of RF-based systems. By explicitly associating attack types with specific wireless technologies, the analysis provides a structured basis for prioritizing security efforts according to the relative exposure of different systems to RF-originated threats.

Although this study is limited to publications indexed in Scopus and Web of Science, the consistency of the observed patterns across technologies and attack classes suggests that the findings offer practical guidance for engineers, system designers, and researchers. In particular, the identified vulnerability profiles can assist in focusing mitigation strategies on RF technologies that exhibit higher exposure and greater potential for cascading impacts in interconnected wireless ecosystems.

### Future Research Directions in RF Cybersecurity

The scientometric and systematic analyses presented in this study indicate that research on cybersecurity in radio frequency technologies has reached a stage of scientific consolidation, particularly with respect to well-known attack vectors such as jamming and spoofing across GNSS, IoT, and wireless communication systems. At the same time, the results reveal several directions in which future research is both necessary and promising, especially as RF-based systems continue to expand in scale, complexity, and criticality.

One important research direction concerns the development of RF-aware security frameworks that explicitly integrate physical-layer characteristics with higher-layer security mechanisms, such as authentication, key management, access control, and intrusion detection. While many existing studies focus on isolated protocol-level or application-level defenses, emerging RF environments, such as dense IoT deployments, smart cities, and cyber–physical systems, require holistic approaches capable of capturing spectrum dynamics, interference patterns, and cross-layer dependencies [5,7,57]. This integration is particularly relevant for low-power and resource-constrained devices, where traditional cryptographic solutions alone may be insufficient or impractical.

Another promising avenue lies in the advancement of adaptive and data-driven detection techniques for RF threats. The increasing use of machine learning and signal intelligence for jamming and spoofing detection reflects a broader shift toward data-centric security models [85,86]. However, the literature still lacks systematic evaluation of these techniques under realistic RF conditions, including heterogeneous devices, non-stationary noise, and adversarial manipulation. Future research should therefore prioritize robustness, explainability, and generalization across different RF technologies and operating conditions, rather than optimizing detection performance only under narrowly controlled or idealized experimental scenarios.

The results also highlight gaps in the security literature for emerging RF technologies, such as 6G and satellite-IoT, as well as for less extensively studied systems like ultra-wideband (UWB). Compared to established platforms such as GNSS, Wi-Fi, and LoRaWAN, these technologies remain under-represented in RF cybersecurity studies [8,150]. This gap points to the need for comparative analyses across RF technologies, studies based on realistic or semi-realistic attack scenarios, and investigations that relate spectrum characteristics to security vulnerabilities, as well as assessments of the broader system-level impact of RF attacks.

Finally, future research should place emphasis on the implications of RF cybersecurity for real-world deployments and critical infrastructures. As demonstrated by the attack–technology mappings presented in this study, RF vulnerabilities can directly affect safety-critical systems, including autonomous vehicles, industrial automation, and public safety communications. Bridging the gap between analytical research and deployable countermeasures, through standardized threat models, cross-technology benchmarks, and interdisciplinary collaboration, remains an open challenge and a key priority for the maturation of the field [6,11].

Taken together, these directions point to the need for studies that compare RF technologies and attack types in a structured way, rather than addressing each technology or vulnerability in isolation.

## 6. Conclusions

This study demonstrates that cybersecurity research in radio frequency technologies has expanded over the past decade and has evolved into two well-defined thematic directions. One is centered on signal integrity and authentication in GNSS and cellular networks, where spoofing and jamming remain dominant concerns; the other focuses on the resilience of IoT and short-range wireless systems, which face a broader set of threats such as replay, relay, eavesdropping, and man-in-the-middle attacks. Together, these directions reflect the growing integration of RF security into discussions on reliability, interoperability, and the protection of cyber–physical infrastructures.

The scientometric analysis highlights the prominence of Sensors and leading IEEE journals as the primary publication venues for RF security research, as well as the strong concentration of scientific production in Asia, particularly China, which has played a decisive role in advancing methods for detecting, mitigating, and modeling RF-based threats. These patterns portray a research domain that is technologically driven, globally connected, and increasingly multidisciplinary.

Despite this growth, important gaps remain. Several widely deployed RF Technologies, such as RFID, NFC, BLE, ZigBee, LoRa, Wi-Fi, and unlicensed ISM bands, still lack comprehensive cybersecurity assessments, especially regarding attack vectors like credential cloning, relay manipulation, and signal injection. Emerging fields, including terahertz communication and 6G systems, are also underexplored from a security perspective, representing promising opportunities for future investigations. In this context, future research should prioritize spectrum-aware threat modeling, experimental validation in real-world RF environments, and the development of adaptive detection mechanisms capable of operating under the scalability and latency constraints of next-generation wireless systems.

Overall, the findings of this study reinforce the importance of continued monitoring of vulnerabilities across established and emerging RF technologies, as well as strengthening international collaboration to keep pace with the rapid evolution of wireless communication ecosystems. By mapping cross-cutting threats and identifying technology-specific exposure patterns, this work provides a practical foundation to support future research and guide the development of more resilient wireless and IoT systems.

## Figures and Tables

**Table 1:** Evolution of prompt refinement and comparative agreement between the LLM and human validation.

Prompt	Description	Sample (*n*)	Agreement (%)	κ
v1	Simple structure, no examples, partial A–B checklist	200	74.5	0.48
v2	Added examples and complete A–B–C checklist	250	82.1	0.63
v3	Introduced semantic rules and hybrid examples to increase sensitivity	300	89.2	0.78
v4 (final)	Semantic refinement and full human-in-the-loop validation with contextual tuning	554	98.6	0.97

**Table 2:** Main information about the dataset.

Description	Results
Timespan	2009–2025
Sources (Journals, Books, etc.)	234
Documents	678
Annual Growth Rate (%)	28.01
Document Average Age	3.65
Average Citations per Document	18.39
References	0
Keywords Plus (ID)	2623
Author’s Keywords (DE)	2728
Authors	1790
Authors of Single-authored Documents	11
Single-authored Documents	11
Co-authors per Document	4.12
International Co-authorships (%)	16.22

**Table 3:** Average citations per year for RF cybersecurity publications (2009–2025).

Year	Mean TC per Art	N	Mean TC per Year	Citable Years
2009	57.00	2	3.35	17
2010	139.00	1	8.69	16
2011	53.25	4	3.55	15
2012	73.43	7	5.24	14
2013	113.33	6	8.72	13
2014	28.67	9	2.39	12
2015	47.79	19	4.34	11
2016	33.24	17	3.32	10
2017	34.21	29	3.80	9
2018	44.00	38	5.50	8
2019	23.72	29	3.39	7
2020	29.06	50	4.84	6
2021	19.32	75	3.86	5
2022	15.82	77	3.96	4
2023	10.38	102	3.46	3
2024	4.60	109	2.30	2
2025	0.45	104	0.45	1

**Table 4:** Top 10 most relevant sources in the field of RF cybersecurity.

Source Title	Number of Articles
MDPI Sensors	41
IEEE Internet of Things Journal	34
IEEE Transactions on Vehicular Technology	32
IEEE Access	31
IEEE Transactions on Aerospace and Electronic Systems	22
IEEE Transactions on Wireless Communications	20
IEEE Wireless Communications Letters	18
IEEE Transactions on Information Forensics and Security	17
IEEE Sensors Journal	16
IEEE Transactions on Dependable and Secure Computing	11

**Table 5:** Top 10 most relevant article authors in RF cybersecurity research.

Authors	Number of Articles	Number of Articles Fractionalized
LI H.	17	4.40
LI Y.	17	3.52
LIU Y.	16	3.88
WANG H.	16	4.04
LIU J.	15	2.87
WANG Y.	15	4.05
LU M.	14	4.03
MOSAVI M.	14	4.95
WANG J.	14	2.60
ZHANG Y.	14	2.51

**Table 6:** Top 10 most relevant affiliations in RF cybersecurity research.

Affiliation	Number of Articles
Xidian University	24
Tsinghua University	22
Beijing Jiaotong University	21
Southeast University—China	21
Nanjing University of Posts and Telecomm.	20
Beihang University	18
Xi’an Jiaotong University	18
Hong Kong Polytechnic University	16
Nanyang Technological University	13
Civil Aviation University of China	12

**Table 7:** Top 10 most cited countries in RF cybersecurity research.

Country	Total Citations (TC)	Average Article Citations
China	4154	16.00
USA	3286	30.70
Italy	448	17.20
Iran	302	12.60
France	283	35.40
Canada	245	20.40
India	237	7.90
United Kingdom	198	16.50
Korea	192	8.70
Australia	136	17.00

**Table 8:** Top 10 most globally cited documents in RF cybersecurity research.

Paper	Total Citations	TC per Year	Normalized TC
Shepard D, 2012, *Int. J. Crit. Infr. Prot.* [110]	211	15.07	2.87
Xiao L, 2018, *IEEE Wirel. Commun.* [111]	201	25.13	4.57
Sankhe K, 2020, *IEEE Trans. Cogn. Commun. Netw.* [112]	194	32.33	6.68
Humphreys T, 2013, *IEEE T. Aero. Elec. Sys.* [113]	184	14.15	1.62
Psiaki M, 2013, *IEEE Trans. Aerosp. Electron. Syst.* [114]	177	13.62	1.56
Jiang X, 2013, *IEEE T. Power Syst.* [115]	174	13.38	1.54
Reising D, 2015, *IEEE Trans. Inf. Forensics Secur.* [116]	162	14.73	3.39
Sedjelmaci H, 2018, *IEEE T. Syst. Man Cybern.* [70]	157	19.63	3.57
He D, 2017, *IEEE Wirel. Commun.-a* [117]	150	16.67	4.39
Guo Y, 2019, *IEEE Trans. Veh. Technol.* [118]	140	20.00	5.90

**Table 9:** Top 10 technology × attack combinations in RF cybersecurity research.

RF Technology	RF Attack Type	Number of Articles
GNSS (GPS/GLONASS/Galileo/BeiDou)	RF Spoofing	245
5G (New Radio)	RF Jamming	53
GNSS (GPS/GLONASS/Galileo/BeiDou)	RF Jamming	49
5G (New Radio)	RF Spoofing	27
6G (emerging)	RF Jamming	23
Wi-Fi (IEEE 802.11 family)	RF Jamming	19
4G/LTE	RF Jamming	17
5G (New Radio)	Eavesdropping	14
LoRa/LoRaWAN	RF Jamming	13
ZigBee (IEEE 802.15.4)	RF Jamming	12

**Table 10:** RF attacks, associated technologies, affected IoT systems, and application domains.

RF Attack Type	RF Technologies	IoT Devices	Application Example	Papers
RF Jamming	GNSS, LoRa/LoRaWAN, 433 MHz, Bluetooth/BLE, RFID, Wi-Fi, ZigBee, UWB, 4G/5G/6G	UAVs, vehicle systems, smart home devices, IoT sensors, RFID systems, industrial IoT nodes, satellite-IoT devices.	Navigation disruption, communication denial, smart-home blocking, industrial interference, flight-safety degradation.	[6,7,8,54,62,89,91,120,122,125,153,154,155,164,165,166,167,168,169,170,171,172,173,174,175,176,177,178,179,180,181,182,183,184]
RF Spoofing	GNSS, LoRa/LoRaWAN, Wi-Fi, 5G/6G	UAVs, autonomous vehicles, GNSS-based sensors, industrial IoT devices.	False-position injection, navigation manipulation, system misdirection, process falsification.	[10,13,66,67,151,169,177,185,186,187,188,189,190]
Eavesdropping	433 MHz, Bluetooth/BLE, RFID, V2V	Smart-home devices, vehicle systems, RFID tags/readers.	Data interception, vehicle-to-vehicle sniffing, credential leakage.	[154,167]
RF Replay Attack	GNSS, LoRa/LoRaWAN, 433 MHz, Bluetooth/BLE, RFID, NB-IoT, Wi-Fi, ZigBee	UAVs, smart locks, IoT sensors/actuators, RFID systems, NB-IoT devices, ZigBee devices.	Access replay, unauthorized unlocking, sensor data reuse, fake status messages.	[6,63,120,154,162,166,172,186,191,192,193]
RF Signal Injection	LoRa/LoRaWAN, UWB, 5G	LoRa end-devices, UWB localization nodes, multi-hop IoT links.	False-signal insertion, positioning manipulation, network misbehavior induction.	[9,135]
Man-in-the-Middle (MitM)	LoRa/LoRaWAN, 433 MHz, Bluetooth/ BLE, RFID	LoRa devices, smart-home systems, RFID infrastructures.	Traffic interception, message alteration, command manipulation.	[154,162,181,194]
RF Credential Cloning	LoRa/LoRaWAN, RFID	LoRa sensors/gateways, RFID tags/readers.	Identity duplication, unauthorized access, device impersonation.	[154,182]

## Data Availability

The review protocol and supplementary methodological materials are publicly available in the Open Science Framework (OSF) at https://osf.io/bqx7n/ (accessed on 11 December 2025).

## Abbreviations

The following abbreviations are used in this manuscript:

4G	Fourth Generation Cellular Network
5G	Fifth Generation Cellular Network
6G	Sixth Generation Cellular Network
BLE	Bluetooth Low Energy
DSRC	Dedicated Short Range Communications
DT	Document Type
GLONASS	Global Orbiting Navigation Satellite System
GNSS	Global Navigation Satellite System
GNU	GNU’s Not Unix
GPS	Global Positioning System
IEEE	Institute of Electrical and Electronics Engineers
IoMT	Internet of Medical Things
IoT	Internet of Things
IP	Internet Protocol
LLM	Large Language Model
LoRa	Long Range
LoRaWAN	Long Range Wide Area Network
LTE	Long-Term Evolution
MCP	Multiple Country Publications
MDPI	Multidisciplinary Digital Publishing Institute
MitM	Man in the Middle
NB-IoT	Narrowband Internet of Things
NFC	Near Field Communication
OSI	Open Systems Interconnection
PRISMA	Preferred Reporting Items for Systematic Reviews and Meta-Analyses
PUEA	Primary User Emulation Attack
QoS	Quality of Service
RAG	Retrieval-Augmented Generation
RF	Radio Frequency
RFID	Radio Frequency Identification
RTL-SDR	Realtek Software-Defined Radio
SCP	Single Country Publications
SDR	Software-Defined Radio
TC	Total Citations
TCP	Transfer Control Protocol
USRP	Universal Software Radio Peripheral
UWB	Ultra-Wideband
V2X	Vehicle-to-Everything

## Appendix A. Search Strategy

### Appendix A.1. General Structure of the Search String

The search string was designed to capture publications addressing the intersection between cybersecurity and radio frequency communication technologies by applying Boolean and semantic proximity operators. The goal was to achieve broad sensitivity while maintaining thematic precision, ensuring the inclusion of articles focused on threats, vulnerabilities, and protection mechanisms in RF communication systems, while excluding topics strictly related to physical-layer or antenna studies (Table A1).

**Table A1:** Search strings applied in Scopus and Web of Science databases.

Database	Search Field	Search String
Scopus	TITLE-ABS-KEY	((((“cyber_securit*” OR cyber) AND (vulnerabilit* OR attack* OR threat*)) OR “jamming attack” OR “replay attack” OR “side-channel attack” OR “sniffing attack” OR “spoofing attack” OR “tampering attack” OR “man-in-the-middle attack” OR “SDR attack” OR “SDR hacking” OR “spectrum sensing attack” OR “spectrum sensing falsification” OR SSDF OR “cognitive radio attack” OR “cognitive radio security”) AND (“radio_frequenc*” OR “wireless communication” OR “Wi-Fi” OR WiFi OR “Wi Fi” OR Bluetooth OR “Bluetooth Low Energy” OR BLE OR ZigBee OR LoRa OR LoRaWAN OR NFC OR RFID OR “ISM band” OR NB_IoT OR “Narrowband IoT” OR LTE_M OR “LTE M” OR “5G” OR “millimeter wave” OR mmWave OR “Z-Wave” OR ZWave OR Sigfox OR Mioty OR “Ultra Wideband” OR UWB OR “3G” OR “4G” OR LTE OR Thread OR 6LoWPAN OR GPS OR GNSS OR “Satellite IoT” OR “Vehicle to Everything” OR V2X OR DSRC OR EnOcean OR “Citizens Broadband Radio Service” OR “Digital Audio Broadcasting” OR “Tire Pressure Monitoring System” OR “Satellite radio” OR Galileo OR GLONASS OR BeiDou OR LPWAN OR “Radar FMCW” OR “Frequency Modulated Continuous Wave” OR “Electronic Toll Collection” OR “Wi-SUN” OR “6.78 MHz” OR “13.56 MHz” OR “27 MHz” OR “40.68 MHz” OR “125 kHz” OR “174 MHz” OR “240 MHz” OR “315 MHz” OR “433 MHz” OR “530 kHz” OR “1710 kHz” OR “868 MHz” OR “915 MHz” OR “1.2 GHz” OR “1.6 GHz” OR “1.45 GHz” OR “2.3 GHz” OR “2.4 GHz” OR “5.8 GHz” OR “5.9 GHz” OR “6 GHz” OR “24 GHz” OR “76 GHz” OR “81 GHz” OR “3.1 GHz” OR “10.6 GHz” OR “100 GHz” OR ((MHz OR kHz OR GHz) W/5 (vulnerabilit* OR attack* OR jamm* OR spoof* OR replay OR “man-in-the-middle” OR “side-channel”))))
Web of Science	Topic Search (TS)	((((“cyber_securit*” OR cyber) AND (vulnerabilit* OR attack* OR threat*)) OR “jamming attack” OR “replay attack” OR “side-channel attack” OR “sniffing attack” OR “spoofing attack” OR “tampering attack” OR “man-in-the-middle attack” OR “SDR attack” OR “SDR hacking” OR “spectrum sensing attack” OR “spectrum sensing falsification” OR SSDF OR “cognitive radio attack” OR “cognitive radio security”) AND (“radio_frequenc*” OR “wireless communication” OR “Wi-Fi” OR WiFi OR “Wi Fi” OR Bluetooth OR “Bluetooth Low Energy” OR BLE OR ZigBee OR LoRa OR LoRaWAN OR NFC OR RFID OR “ISM band” OR NB_IoT OR “Narrowband IoT” OR LTE_M OR “LTE M” OR “5G” OR “millimeter wave” OR mmWave OR “Z-Wave” OR ZWave OR Sigfox OR Mioty OR “Ultra Wideband” OR UWB OR “3G” OR “4G” OR LTE OR Thread OR 6LoWPAN OR GPS OR GNSS OR “Satellite IoT” OR “Vehicle to Everything” OR V2X OR DSRC OR EnOcean OR “Citizens Broadband Radio Service” OR “Digital Audio Broadcasting” OR “Tire Pressure Monitoring System” OR “Satellite radio” OR Galileo OR GLONASS OR BeiDou OR LPWAN OR “Radar FMCW” OR “Frequency Modulated Continuous Wave” OR “Electronic Toll Collection” OR “Wi-SUN” OR “6.78 MHz” OR “13.56 MHz” OR “27 MHz” OR “40.68 MHz” OR “125 kHz” OR “174 MHz” OR “240 MHz” OR “315 MHz” OR “433 MHz” OR “530 kHz” OR “1710 kHz” OR “868 MHz” OR “915 MHz” OR “1.2 GHz” OR “1.6 GHz” OR “1.45 GHz” OR “2.3 GHz” OR “2.4 GHz” OR “5.8 GHz” OR “5.9 GHz” OR “6 GHz” OR “24 GHz” OR “76 GHz” OR “81 GHz” OR “3.1 GHz” OR “10.6 GHz” OR “100 GHz” OR ((MHz OR kHz OR GHz) NEAR/5 (vulnerabilit* OR attack* OR jamm* OR spoof* OR replay OR “man-in-the-middle” OR “side-channel”))))

### Appendix A.2. Lists of Thematic Descriptors

(a) Cybersecurity and Threat Terms
  - cybersecurity, cyber attack, vulnerability, threat;
  - jamming, spoofing, replay, relay, eavesdropping, sniffing;
  - man-in-the-middle, injection, denial of service, DoS, IDS, encryption, authentication;
  - spectrum sensing falsification (SSDF), cognitive radio attack, side-channel attack.
(b) RF Communication Technologies and Protocols
  - Short-range networks: Bluetooth, BLE, ZigBee, RFID, NFC, EnOcean;
  - IoT and LPWAN networks: LoRa, LoRaWAN, Sigfox, Mioty, NB-IoT, LTE-M, Z-Wave, Thread, 6LoWPAN, Wi-SUN;
  - Wide-area networks: Wi-Fi (IEEE 802.11), 3G, 4G, LTE, 5G;
  - Positioning and satellite technologies: GPS, GNSS, Galileo, GLONASS, BeiDou, Satellite IoT, Satellite Radio;
  - Critical applications: V2X, DSRC, Radar FMCW, Electronic Toll Collection, Tire Pressure Monitoring System.
(c) Frequency Bands
  - Low and mid-frequency bands: 125 kHz, 13.56 MHz, 27 MHz, 40.68 MHz, 315 MHz, 433 MHz, 868 MHz, 915 MHz;
  - Upper frequency bands: 2.3–2.4 GHz, 5.8–5.9 GHz, 6 GHz, 24 GHz, 76–81 GHz;
  - Broadband bands: 3.1–10.6 GHz (UWB), 24–100 GHz (mmWave).

## Appendix B. PRISMA 2020 Checklist

This appendix presents the complete PRISMA 2020 checklist used to guide the reporting of this systematic review. Each item is mapped to the corresponding section, subsection, figure, or table in the manuscript where the requirement is addressed. The checklist is provided to enhance transparency, reproducibility, and methodological rigor, and to facilitate verification of compliance with the PRISMA 2020 reporting guidelines.

**Table A2:** PRISMA 2020 checklist for the systematic review.

Section and Topic	Item #	Checklist Item	Location Where Item Is Reported
TITLE	1	Identify the report as a systematic review.	p. 1
ABSTRACT	2	See the PRISMA 2020 for Abstracts checklist.	p. 1
INTRODUCTION	3	Describe the rationale for the review in the context of existing knowledge.	Section 1, pp. 1–2
INTRODUCTION	4	Provide an explicit statement of the objective(s) or question(s) the review addresses.	Section 1, p. 2–3
METHODS	5	Specify the inclusion and exclusion criteria for the review and how studies were grouped for the syntheses.	Section 2.1, pp. 4–5 and Section 2.3, p. 7
METHODS	6	Specify all databases, registers, websites, organisations, reference lists and other sources searched or consulted to identify studies. Specify the date when each source was last searched or consulted.	Section 2.1, p. 4
METHODS	7	Present the full search strategies for all databases, registers and websites, including any filters and limits used.	Section 2.1, pp. 4–5
METHODS	8	Specify the methods used to decide whether a study met the inclusion criteria of the review, including how many reviewers screened each record and each report retrieved, whether they worked independently, and, if applicable, details of automation tools used in the process.	Section 2.2 and Section 2.3, Figure 2 and Figure 3
METHODS	9	Specify the methods used to collect data from reports, including how many reviewers collected data from each report, whether they worked independently, any processes for obtaining or confirming data from study investigators, and, if applicable, details of automation tools used in the process.	Section 2.1, pp. 4–5 and Section 2.3, pp. 7–9
METHODS	10a	List and define all outcomes for which data were sought. Specify whether all results compatible with each outcome domain in each study were sought (e.g., for all measures, time points, analyses) and, if not, the methods used to decide which results to collect.	Section 2.3, p. 8
METHODS	10b	List and define all other variables for which data were sought (e.g., participant and intervention characteristics, funding sources). Describe any assumptions made about any missing or unclear information.	Section 2.3, p. 8
METHODS	11	Specify the methods used to assess risk of bias in the included studies, including details of the tool(s) used, how many reviewers assessed each study and whether they worked independently, and, if applicable, details of automation tools used in the process.	Section 2.4, p. 9
METHODS	12	Specify for each outcome the effect measure(s) (e.g., risk ratio, mean difference) used in the synthesis or presentation of results.	Section 3.1, p. 10 —Table 2
METHODS	13a	Describe the processes used to decide which studies were eligible for each synthesis (e.g., tabulating the study characteristics and comparing against the planned groups for each synthesis).	Section 2.2, pp. 5–7, Section 2.4, pp. 9–10 and Section 3.1, p. 10
METHODS	13b	Describe any methods required to prepare the data for presentation or synthesis (e.g., handling missing summary statistics, data conversions).	Section 2.2, pp. 5–7, Section 2.4, pp. 9–10 and Section 3.1, p. 10
METHODS	13c	Describe any methods used to tabulate or visually display results of individual studies and syntheses.	Section 2.1, pp. 4–5 and Section 3, pp. 10–23
METHODS	13d	Describe any methods used to synthesize results and provide a rationale for the choice(s). If meta-analysis was performed, describe the model(s), method(s) to identify statistical heterogeneity, and software package(s) used.	Section 2, pp. 3–7 and Section 3, pp. 10–15. No meta-analysis was performed; synthesis is narrative and scientometric.
METHODS	13e	Describe any methods used to explore possible causes of heterogeneity among study results (e.g., subgroup analysis, meta-regression).	Not applicable; no quantitative outcomes or study-level effect estimates were extracted; therefore, no heterogeneity analysis was conducted.
METHODS	13f	Describe any sensitivity analyses conducted to assess robustness of the synthesized results.	Not applicable; the review does not include quantitative synthesis or effect estimates, so no sensitivity analyses were required or performed.
METHODS	14	Describe any methods used to assess risk of bias due to missing results in a synthesis (arising from reporting biases).	Section 2.4, p. 9. Note: reporting bias was not formally assessed using a specific tool; however, human-in-the-loop validation and reclassification procedures were applied to reduce misclassification bias.
METHODS	15	Describe any methods used to assess certainty (or confidence) in the body of evidence for an outcome.	Not formally assessed; certainty of evidence was not evaluated using a standardized framework, as the review is scientometric and descriptive.
RESULTS	16a	Describe the results of the search and selection process, from the number of records identified to the number of studies included, ideally using a flow diagram.	Section 2.2, pp. 5–7 —Figure 2
RESULTS	16b	Cite studies that might appear to meet the inclusion criteria, but which were excluded, and explain why they were excluded.	Section 2.2 and Section 2.3, pp. 5–9, where categories and reasons for exclusion are described. Individual excluded studies are not listed.
RESULTS	17	Cite each included study and present its characteristics.	Section 4, pp. 24–28
RESULTS	18	Present assessments of risk of bias for each included study.	Not applicable; the review did not assess risk of bias of individual studies because the objective was scientometric and descriptive, and included publications were not evaluated in terms of methodological rigor or internal validity.
RESULTS	19	For all outcomes, present, for each study: (a) summary statistics for each group (where appropriate) and (b) an effect estimate and its precision (e.g., confidence/credible interval), ideally using structured tables or plots.	Not applicable; no individual effect sizes, experimental outcomes, or statistical estimates were extracted. The review is descriptive and scientometric; therefore, individual study results are not presented. General characteristics are summarised in Table 1 (p. 10).
RESULTS	20a	For each synthesis, briefly summarise the characteristics and risk of bias among contributing studies.	Section 3 (Results) and Table 2 (p. 10); no formal risk-of-bias tool was applied.
RESULTS	20b	Present results of all statistical syntheses conducted. If meta-analysis was done, present the summary estimate and its precision and measures of heterogeneity.	Not applicable; no meta-analysis or statistical synthesis was performed. Results are presented through descriptive statistics and scientometric indicators (Section 3 and Table 2 (p. 10)).
RESULTS	20c	Present results of all investigations of possible causes of heterogeneity among study results.	Not applicable; no meta-analysis or formal certainty/reporting bias assessment was performed due to the descriptive and scientometric nature of the review.
RESULTS	20d	Present results of all sensitivity analyses conducted to assess the robustness of the synthesized results.	Not applicable; no meta-analysis or formal certainty/reporting bias assessment was performed due to the descriptive and scientometric nature of the review.
RESULTS	21	Present assessments of risk of bias due to missing results (arising from reporting biases) for each synthesis assessed.	Not applicable; no meta-analysis or formal certainty/reporting bias assessment was performed due to the descriptive and scientometric nature of the review.
RESULTS	22	Present assessments of certainty (or confidence) in the body of evidence for each outcome assessed.	Not applicable; no meta-analysis or formal certainty/reporting bias assessment was performed due to the descriptive and scientometric nature of the review.
DISCUSSION	23a	Provide a general interpretation of the results in the context of other evidence.	Section 5, pp. 28–30
DISCUSSION	23b	Discuss any limitations of the evidence included in the review.	Section 5, pp. 28–30
DISCUSSION	23c	Discuss any limitations of the review processes used.	Section 5, pp. 28–30
DISCUSSION	23d	Discuss implications of the results for practice, policy, and future research.	Section 5, pp. 28–30
OTHER INFORMATION	24a	Provide registration information for the review, including register name and registration number, or state that the review was not registered.	The review protocol was registered in the Open Science Framework (OSF): https://osf.io/bqx7n (accessed on 11 December 2025).
OTHER INFORMATION	24b	Indicate where the review protocol can be accessed, or state that a protocol was not prepared.	p. 31
OTHER INFORMATION	24c	Describe and explain any amendments to information provided at registration or in the protocol.	No amendments were made to the registered protocol.
OTHER INFORMATION	25	Describe sources of financial or non-financial support for the review, and the role of the funders or sponsors in the review.	p. 31
OTHER INFORMATION	26	Declare any competing interests of review authors.	p. 31
OTHER INFORMATION	27	Report which materials are publicly available and where they can be found (e.g., forms, extracted data, analytic code, other materials).	p. 31. The review protocol, the minimal documented RF triage script, and the final deduplicated dataset used for screening are publicly available at the OSF repository https://osf.io/bqx7n (accessed on 11 December 2025). Raw Scopus and Web of Science exports cannot be shared due to licensing restrictions.

## Appendix C. Computation of Agreement Metrics

This appendix details the computation of the observed agreement ($p_{o}$) and the expected agreement by chance ($p_{e}$) used to calculate Cohen’s kappa coefficient reported in Section 2.4. The purpose of this appendix is to ensure full methodological transparency and reproducibility of the reliability analysis.

### Appendix C.1. Validation Sample and Decision Framework

The human-in-the-loop validation was performed on a stratified sample of n=554 articles, composed of:

- 150 articles initially classified as *YES*;
- 300 articles initially classified as *NO*;
- 104 articles initially classified as *MAYBE*.

In this study, the *MAYBE* category was treated as a hybrid inclusion class. All articles labeled as *MAYBE* by the LLM were subjected to full human review. When a direct relationship between cybersecurity and radio frequency communication was confirmed, these articles were reclassified as *YES*. Consequently, the final agreement analysis was conducted in a binary decision space (*YES* / *NO*), which is consistent with common practices in systematic screening and validation studies.

After manual validation, 96 of the 104 *MAYBE* articles were confirmed as relevant and incorporated into the *YES* category, while the remaining 8 were excluded.

### Appendix C.2. Observed Agreement ($p_{o}$)

The observed agreement ($p_{o}$) represents the proportion of cases in which the LLM classification was consistent with the final human decision. Agreement was counted in the following situations:

- Articles classified as *YES* by both the LLM and human reviewers (n=150);
- Articles classified as *NO* by both the LLM and human reviewers (n=300);
- Articles initially classified as *MAYBE* by the LLM and subsequently validated as *YES* after human review (n=96).

Thus, the total number of concordant decisions was:

150+300+96=546

Considering the total validation sample of n=554 articles, the observed agreement was calculated as:

$$p_{o}=\frac{546}{554}=0.986$$

### Appendix C.3. Expected Agreement by Chance ($p_{e}$)

The expected agreement by chance ($p_{e}$) was computed from the marginal class proportions of the final binary labels (*YES* vs. *NO*). In this study, the intermediate category (*MAYBE*) was not treated as an independent class in the kappa computation. Instead, all *MAYBE* cases were resolved through the human-in-the-loop (HITL) validation process and mapped to a definitive inclusion decision (*YES* or *NO*). Consequently, the final Cohen’s kappa was calculated under a binary decision framework, reflecting agreement on the final inclusion outcome after human validation.

Let $P_{\mathrm{YES}}^{\mathrm{LLM}}$ and $P_{\mathrm{NO}}^{\mathrm{LLM}}$ denote the marginal proportions of the final classifications produced by the LLM after HITL resolution, and $P_{\mathrm{YES}}^{H}$ and $P_{\mathrm{NO}}^{H}$ the corresponding proportions obtained from human validation. All proportions were computed on the validation sample (n=554).

Human (final) distribution.

After human validation, 246 articles were considered relevant (*YES*), while 308 were excluded (*NO*), yielding:

$$P_{\mathrm{YES}}^{H}=\frac{246}{554}=0.444,\;P_{\mathrm{NO}}^{H}=\frac{308}{554}=0.556$$

LLM (final) distribution.

In the final accounting, the LLM *YES* category comprises the 150 articles initially classified as *YES* and confirmed by human review, plus the 96 articles initially labeled as *MAYBE* that were subsequently validated as relevant. The remaining 308 articles were classified as *NO* after validation. Therefore:

$$P_{\mathrm{YES}}^{\mathrm{LLM}}=\frac{150+96}{554}=\frac{246}{554}=0.444,\;P_{\mathrm{NO}}^{\mathrm{LLM}}=\frac{308}{554}=0.556$$

The expected agreement by chance was then computed as:

$$p_{e}=P_{\mathrm{YES}}^{\mathrm{LLM}}\cdot P_{\mathrm{YES}}^{H}+P_{\mathrm{NO}}^{\mathrm{LLM}}\cdot P_{\mathrm{NO}}^{H}$$

$$p_{e}=\left(0.444\cdot 0.444\right)+\left(0.556\cdot 0.556\right)$$

$$p_{e}=0.197+0.309=0.506$$

This value represents the level of agreement that would be expected by chance alone under the final binary classification scheme adopted in this study, after resolving all intermediate *MAYBE* cases through human validation.

### Appendix C.4. Cohen’s Kappa Coefficient

Substituting the computed values of $p_{o}$ and $p_{e}$ into Equation 1, Cohen’s kappa coefficient was obtained as:

$$\kappa =\frac{p_{o}-p_{e}}{1-p_{e}}=\frac{0.986-0.506}{1-0.506}=0.97$$

According to the interpretation scale proposed by [49], a kappa value above 0.81 indicates an *almost perfect* level of agreement. This result confirms the stability and reliability of the LLM-assisted semantic screening after supervised prompt refinement, and supports the robustness of the final corpus used in the scientometric analysis.
